# NLRP7 deubiquitination by USP10 promotes tumor progression and tumor-associated macrophage polarization in colorectal cancer

**DOI:** 10.1186/s13046-021-01920-y

**Published:** 2021-04-10

**Authors:** Bing Li, Zhi-Peng Qi, Dong-Li He, Zhang-Han Chen, Jing-Yi Liu, Meng-Wai Wong, Jia-Wei Zhang, En-Pan Xu, Qiang Shi, Shi-Lun Cai, Di Sun, Li-Qing Yao, Ping-Hong Zhou, Yun-Shi Zhong

**Affiliations:** 1 Endoscopy Center, Zhongshan Hospital of Fudan University, 180 Fenglin Road, Shanghai, 20032 People’s Republic of China; 2 Endoscopy Research Institute of Fudan University, Shanghai, 20032 People’s Republic of China; 3 Endoscopy Center, Xuhui Hospital, Zhongshan Hospital of Fudan University, Shanghai, 20031 People’s Republic of China; 4 Department of Gastroenterology, Xuhui Hospital, Zhongshan Hospital of Fudan University, Shanghai, 20031 People’s Republic of China

**Keywords:** Colorectal cancer, NOD-like receptors, NLRP7, Deubiquitination, Tumor-associated macrophages

## Abstract

**Background:**

NOD-like receptors affect multiple stages of cancer progression in many malignancies. NACHT, LRR, and PYD domain-containing protein 7 (NLRP7) is a member of the NOD-like receptor family, although its role in tumorigenesis remains unclear. By analyzing clinical samples, we found that NLRP7 protein levels were upregulated in colorectal cancer (CRC). We proposed the hypothesis that a high level of NLRP7 in CRC may promote tumor progression. Here, we further investigated the role of NLRP7 in CRC and the underlying mechanism.

**Methods:**

NLRP7 expression in human CRC and adjacent non-tumorous tissues was examined by quantitative real-time polymerase chain reaction (qRT-PCR), western blotting, and immunohistochemistry. The effect of NLRP7 in CRC progression was investigated in vitro and in vivo. Proteins interacting with NLRP7 were identified by immunoprecipitation and mass spectrometry analysis while immunofluorescence staining revealed the cellular location of the proteins. Cellular ubiquitination and protein stability assays were applied to demonstrate the ubiquitination effect on NLRP7. Cloning and mutagenesis were used to identify a lysine acceptor site that mediates NLRP7 ubiquitination. Cytokines/chemokines affected by NLRP7 were identified by RNA sequencing, qRT-PCR, and enzyme-linked immunosorbent assay. Macrophage phenotypes were determined using qRT-PCR, flow cytometry, and immunohistochemistry.

**Results:**

NLRP7 protein levels, but not mRNA levels, were upregulated in CRC, and increased NLRP7 protein expression was associated with poor survival. NLRP7 promoted tumor cell proliferation and metastasis in vivo and in vitro and interacted with ubiquitin-specific protease 10, which catalyzed its deubiquitination in CRC cells. NLRP7 stability and protein levels in CRC cells were modulated by ubiquitination and deubiquitination, and NLRP7 was involved in the ubiquitin-specific protease 10 promotion of tumor progression and metastasis in CRC. K379 was an important lysine acceptor site that mediates NLRP7 ubiquitination in CRC cells. In CRC, NLRP7 promoted the polarization of pro-tumor M2-like macrophages by inducing the secretion of C-C motif chemokine ligand 2. Furthermore, NLRP7 promoted NF-κB nuclear translocation and activation of C-C motif chemokine ligand 2 transcription.

**Conclusions:**

We showed that NLRP7 promotes CRC progression and revealed an as-yet-unidentified mechanism by which NLRP7 induces the polarization of pro-tumor M2-like macrophages. These results suggest that NLRP7 could serve as a biomarker and novel therapeutic target for the treatment of CRC.

**Supplementary Information:**

The online version contains supplementary material available at 10.1186/s13046-021-01920-y.

## Background

Colorectal cancer (CRC) is one of the most lethal carcinomas, and its incidence is increasing worldwide [1]. Despite an increase in the five-year net survival rates over the past few decades, the mortality rate of CRC remains high, which is mainly due to recurrence and distant organ metastasis [2, 3]. Therefore, exploring the molecular mechanisms that drive tumor initiation and progression in CRC is critical for developing novel preventive and therapeutic strategies against this disease.

NOD-like receptors (NLRs), which were originally studied as critical regulators of the immune response, serve as pattern recognition receptors that specifically recognize pathogen-associated molecular patterns (PAMPs) [4]. Recent evidence supports the concept that innate immune signaling plays an important role in cancer progression [5, 6]. A significant role for members of the NLR family has been revealed as contributing either directly or indirectly to a variety of traits associated with cancer, including inflammation, cell death, tumor growth, angiogenesis, invasion, and metastasis [7–10]. In terms of functionality, some members can be initiators of the inflammasome pathway, which canonically activates caspase-1, and IL-1β and IL-18 thereafter [11, 12]. However, some other NLRs regulate diverse biological pathways associated with both inflammation and tumorigenesis through a non-inflammasome pathway [13–15]. For example, the NLRP3 inflammasome suppresses liver metastasis in CRC by IL-18 signaling [16]. However, NLRP3 upregulation in CRC cells can also promote epithelial-mesenchymal transition in an inflammasome-independent manner [15]. Therefore, in view of the multiple effects of NLR family members in cancer cells, a comprehensive understanding is needed of the multiple signaling pathways in which they are involved.

Among all members of the NLR family, NLRP7 and its function are poorly understood. Previous studies of NLRP7 have investigated effects on trophoblast lineage differentiation and contributions to hydatidiform mole development in abnormal human pregnancies [17, 18]. In a new area of research, a correlation between the *NLRP7* gene and ulcerative colitis (UC) was recently reported, and NLRP7 expression was found to be upregulated in the intestinal mucosa of patients with inflammatory bowel disease (IBD) [19, 20]. Considering that IBD patients have an increased risk of CRC and the function of NLRP7 in cancers remains unclear [21], we decided to explore the contribution of NLRP7 to colorectal tumorigenesis.

In this study, we found that NLRP7 protein levels, but not mRNA levels, were upregulated in CRC by analyzing clinical samples. We then proposed the hypothesis that a high level of NLRP7 in CRC may promote tumor progression, requiring further investigation. The function of NLRP7 in promoting tumor cell proliferation and metastasis was confirmed. Furthermore, we found that NLRP7 in CRC cells interacted with ubiquitin-specific protease 10 (USP10), which mediated its deubiquitination, thereby increasing its protein stability. In addition, NLRP7 promoted the polarization of pro-tumor M2-like macrophages in CRC by inducing the secretion of C-C motif chemokine ligand 2 (CCL2) through the activation of the NF-κB signaling pathway. Thus, we demonstrated that NLRP7 can promote both tumor progression and tumor-associated macrophage polarization in colorectal cancer.

## Methods

### Cell culture and reagents

HCT116 and HT29 cells were cultured in McCoy’s 5A medium (GibCo) supplemented with 10% FBS. DLD-1 and THP-1 cells were grown in RPMI 1640 (GibCo) with 10% FBS. LoVo, SW480, and SW620 cells were cultured in DMEM (GibCo) high-glucose medium with 10% FBS. All cell lines were purchased from Chinese Academy of Sciences Cell Bank (Shanghai, China). THP-1 cells were differentiated into macrophages by exposure to 200 ng/ml phorbol-12-myristate-13-acetate (PMA) (Sigma-Aldrich) for 48 h [22].

### Human tissue specimens and immunohistochemical analysis

Thirty pairs of fresh primary CRC and corresponding non-tumorous tissues were collected immediately after surgical resection at Zhongshan Hospital, Fudan University (Shanghai, China). Another 115 formalin-fixed and paraffin-embedded (FFPE) CRC samples and adjacent non-tumorous tissue samples were also obtained from Zhongshan Hospital. Written informed consent was obtained from each patient, and the investigation was approved by the Ethics Committee of Zhongshan Hospital. Tumor-node-metastasis (TNM) staging was performed according to American Joint Committee on Cancer (AJCC) standards [23].

Consecutive sections of FFPE tumors were subjected to immunohistochemistry (IHC) staining using rabbit polyclonal anti-NLRP7 (Abcam, 1:50) and anti-USP10 (Abcam, 1:50) antibodies. A DAB substrate kit was used according to the manufacturer’s instructions. The results were scored by two pathologists with no prior knowledge of patient characteristics. Corresponding to the percentage of immune-reactive tumor cells (0, 1–5, 6–25, 26–75, and 76–100%, respectively), the staining extent score was on a scale of 0–4. The staining intensity was scored as negative (score = 0), weak (score = 1), medium (score = 2), or strong (score = 3). By multiplying the staining extent score by the intensity score, a score ranging from 0 to 12 was calculated.

### Lentiviral-mediated NLRP7 overexpression and knockdown

A lentiviral construct carrying *NLRP7* (GeneChem, China) was packaged in 293FT cells. Virus-containing supernatants were collected and stably transfected into SW480 and HT29 cells selected by puromycin (Sigma-Aldrich). Empty vector transfected cells were used as controls. Two lentiviral constructs containing short hairpin RNAs (shRNA) were purchased from GeneChem to specifically establish *NLRP7* knockdown cell lines. The sh*NLRP7* construct or the negative control plasmid was transfected into the 293FT cell line, and virus-containing supernatants were collected for subsequent transduction into HCT116 cells and DLD-1 cells. Puromycin (Sigma-Aldrich) was used to select for stably transduced cells. The sequences of the two shRNAs against *NLRP7* are as follows: 5′-TTGCTGAAGAGGAAGATGTTA-3′ and 5′-TGGCAAGAAACTGGCAGAAAT-3′.

### RNA isolation and quantitative real-time PCR (qRT-PCR)

Total RNA was extracted using the TRIzol reagent (Invitrogen), and complementary DNA (cDNA) was synthesized using a reverse transcription-PCR kit (Roche) according to the manufacturer’s instructions. qRT-PCR was performed with Maxima SYBR Green qPCR Master Mix (Applied Biosystems) on a QuantStudio 6 Flex Real-Time PCR System (Applied Biosystems). GAPDH was used as an internal control. The primers were purchased from Asia-Vector Biotechnology Company (Shanghai, China), and their sequences are listed in Additional file 1: Table S1.

### RNA sequencing

After total RNA extraction from DLD-1 and SW480 cells, the RNA quality and quantity were assessed by Nano Drop and Agilent 2100 bioanalyzer (Thermo Fisher Scientific). Strand-specific library construction and sequencing of ~ 100 M paired end.

100-bp-long reads were performed at the BGI (Huada Genomics Institute Co. Ltd., Guangzhou, China). Differential expression analysis was performed using DESeq2 software [24]. GO and KEGG enrichment analysis of annotated differently expressed genes was performed by phyper based on hypergeometric test.

### Western blot (WB) analysis

Quantified protein lysates were separated by sodium dodecyl sulfate-polyacrylamide gel electrophoresis (SDS-PAGE) and transferred onto polyvinylidene difluoride membranes. Subsequently, the proteins were blocked with 5% bovine serum albumin for 1 h at room temperature and incubated in primary antibodies at 4 °C overnight. After washing with TBS-T, the membranes were incubated in horseradish peroxidase-conjugated secondary antibody for 2 h. GAPDH and β-actin were used as loading controls. The antibodies used are listed in Additional file 1: Table S2, and unprocessed original scans of blots are provided in Additional file 2.

### Immunoprecipitation (IP) assay

As described elsewhere [25], whole-cell extracts were prepared in modified buffer (50 mM Tris-HCl pH 7.5, 150 mM NaCl, 5 mM EDTA, and protease inhibitor cocktail) and incubated with the indicated antibodies or IgG at 4 °C for 2 h, and then mixed with 100 μl protein A/G agarose (Santa Cruz) overnight at 4 °C. Beads were washed with PBS three times, and bound protein was denatured with 1× SDS sample buffer. Then, proteins were collected and analyzed by SDS-PAGE and western blotting.

### Mass spectrometry analysis

Similar to the IP assay, cellular protein extracts from HT29 cells were incubated with anti-Flag-NLRP7 followed by and protein A/G agarose beads. Recovered proteins associated with Flag-NLRP7 or IgG were resolved by gel electrophoresis. The bands specifically bind to Flag-NLRP7 were excised, and proteomics screening was accomplished by mass spectrometry analysis on a MALDI-TOF-MS instrument (Bruker Daltonics).

### Immunofluorescence (IF) staining

Immunofluorescent staining was performed as described previously [26]. Briefly, cells were grown on glass-bottom culture dishes until the experimental endpoint. Then, the cells were fixed with 4% paraformaldehyde, then washed three times with PBS, and probed with primary antibody against NLRP7, USP10, or NF-κB p65 at 4 °C overnight. After washing with PBS, the cells were incubated with fluorescence-conjugated secondary antibody (Invitrogen) for 30 min. Next, the cells were washed twice with blocking solution, and once with PBS. Anti-fade DAPI solution (Life Technology) was then added for DNA staining. The cells were imaged using a confocal laser-scanning microscope. Images were analyzed for co-localization using CoLocalizer Pro Version 3.0.2 according to published protocols.

### Cloning and mutagenesis

Full-length human NLRP7 cDNA and truncated NLRP7 were amplified from a human mRNA pool generated by RT-PCR. Site-directed mutagenesis (KKK275RRR, KRK288RRR, K374R, K379R, K461R, K484R, K502R, and K532R) was performed using the Site-Directed Mutagenesis kit (Agilent) according to the manufacturer’s protocol. The sequences of all primers are available in Additional file 1: Table S3. After cloning, cDNA was ligated into the hU6-MCS-CBh-gcGFP-IRES-puromycin vector purchased from GeneChem. The sequences were confirmed by direct sequencing.

### Flow cytometry

Cells were collected and incubated at 4 °C with florescence-conjugated antibodies, and fixed and permeabilized with a fixation/permeabilization solution kit (BD Cytofix/Cytoperm) and 1% paraformaldehyde (PFA). Flow cytometry results were analyzed with FlowJo software. Antibodies used are listed in Additional file 1: Table S2.

### Protein stability assay

In different cell treatment groups, cycloheximide was added into culture medium and cell lysis was collected at 0, 2, 4, 8, 12, 16, or 24 h after the treatment of cycloheximide. Then, the protein was visualized using immunoblotting assays.

### Cellular ubiquitination assay

As described previously, the cellular ubiquitination assays were conducted [27]. Briefly, IP assay was used and the immunoprecipitates were obtained by incubating cell lysates with anti-FLAG-NLRP7 and A/G agarose beads. The ubiquitinated FLAG-NLRP7 was visualized using immunoblotting assays with anti-HA.

### Enzyme linked immunosorbent assay (ELISA)

The supernatant from CRC cells and serum from an animal model of metastasis was collected to detect the secretion level of CCL2 using an ELISA kit (R&D Systems). ELISA was performed according to the manufacturer’s instructions, and all experiments were performed in triplicate.

### Animal studies

The animal experiment was performed with protocols approved by the Institutional Animal Care and Use Committee of Zhongshan Hospital of Fudan University. For in vivo tumorigenic experiments, HCT116 cells (1 × 10^6^) expressing sh-Ctrl, sh-NLRP7, sh-USP10, or sh-USP10/NLRP7 were injected into the right dorsal flank of five-week-old Balb/c nude mice. Tumor formation in nude mice was monitored over a six-week period. The tumor volume was calculated with the following formula: tumor volume = 0.5 × length × width^2^. For in vivo metastasis assays, a small left abdominal flank incision was made, the spleen was exteriorized, and the prepared cells (2 × 10^6^ cells) were injected into the spleen with a 30-Gauge needle. Then, the injected spleen was returned to the abdomen and the wound was sutured with 6–0 black silk. Six weeks later, all mice were sacrificed and necropsied for observation of visible metastatic lesions in the liver. In the metastasis animal assay, the peripheral blood of mice was collected at necropsy and stored at − 80 °C until analysis. All primary and metastatic tumor tissues were harvested for further analysis by IHC staining.

### Statistical analysis

Differences between groups were calculated using Student’s t-test, the chi-square test, or the Fisher exact test. The probability of differences in overall survival (OS) was ascertained by the Kaplan–Meier method with a log-rank test for significance. Analyses were performed with SPSS version 22.0 and Graphpad Prism 7.0 statistical analysis software. Representative data are shown as the mean ± SD. *P* < 0.05 was considered statistically significant.

## Results

### NLRP7 protein with high expression level in CRC promotes tumor cell proliferation and metastasis

**Fig. 1:**
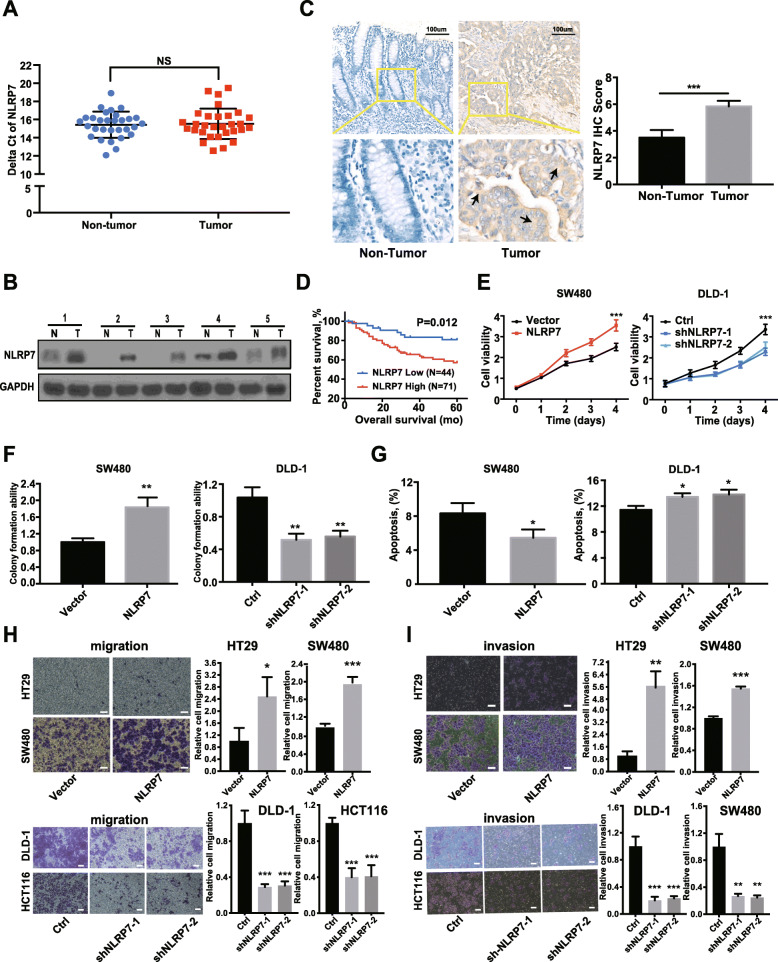
NLRP7 protein with high expression level in CRC promotes tumor cell proliferation and metastasis. **a** NLRP7 mRNA levels in CRC tissues and the normal colorectal mucosa measured by qRT-PCR. **b** The protein levels of NLRP7 in CRC tissues and paired normal tissues measured by WB. N: non-tumor, T: tumor. **c** NLRP7 protein in CRC samples and corresponding adjacent normal tissues confirmed by IHC staining. Scale bars, 100 μm. **d** Kaplan–Meier survival analysis of patients with CRC according to NLRP7 expression (low: score < 6, high: score ≥ 6). Knockdown and overexpression of NLRP7 affected CRC cells proliferation as determined by (**e**) CCK8 assay, (**f**) colony formation ability and (**g**) cell apoptosis. The results of transwell assay of the (**h**) migration and (**i**) invasion for NLRP7 knockdown and overexpression CRC cells. Scale bars, 100 μm. ***P < 0.001, **P < 0.01, and *P < 0.05. NS: no significance

NLRP7 mRNA levels did not differ significantly between 30 CRC tissues and normal colorectal mucosa by qRT-PCR (Fig. 1a), and was confirmed by analysis of gene expression from the GEO dataset (GSE23878, GSE32323, GSE110223, GSE156355) (Additional file 3: Fig. S1A). However, the protein levels of NLRP7 were significantly higher in human CRC tissues than in paired normal tissues as determined by western blotting (Fig. 1b) and confirmed by IHC staining in samples from 115 CRC patients compared with the corresponding adjacent normal tissues (ANTs) (Fig. 1c). The protein expression of NLRP7 in the CRC cohort was significantly correlated with distant metastasis (*P* = 0.004) and advanced clinical stage (*P* = 0.033) (Additional file 1: Table S4). Moreover, Kaplan–Meier survival curve analysis demonstrated that CRC patients with increased NLRP7 protein expression had poorer overall five-year survival (Fig. 1d).

Next, we investigated the biological role of NLRP7 in CRC using cell functional assays. Knockdown of NLRP7 significantly decreased DLD-1 cell proliferation compared with that in the negative control, whereas NLRP7 overexpression significantly increased SW480 cell proliferation (Fig. 1e and f, Additional file 3: Fig. S1B). In addition, the proportion of apoptotic cells decreased in SW480 cells overexpressing NLRP7 and increased in DLD-1 cells after knockdown of NLRP7 (Fig. 1g, Additional file 3: Fig. S1C). To determine the role of NLRP7 in CRC metastasis, we performed migration and invasion assays in vitro. The results showed that overexpression of NLRP7 promoted the migration and invasion of SW480 and HT29 cells, whereas NLRP7 knockdown in DLD-1 and HCT116 cells had the opposite effects (Fig. 1h and i). Collectively, these results indicated that upregulated NLRP7 protein in CRC promotes tumor cell proliferation and metastasis.

### USP10 interacts with NLRP7 and is highly expressed in CRC

**Fig. 2:**
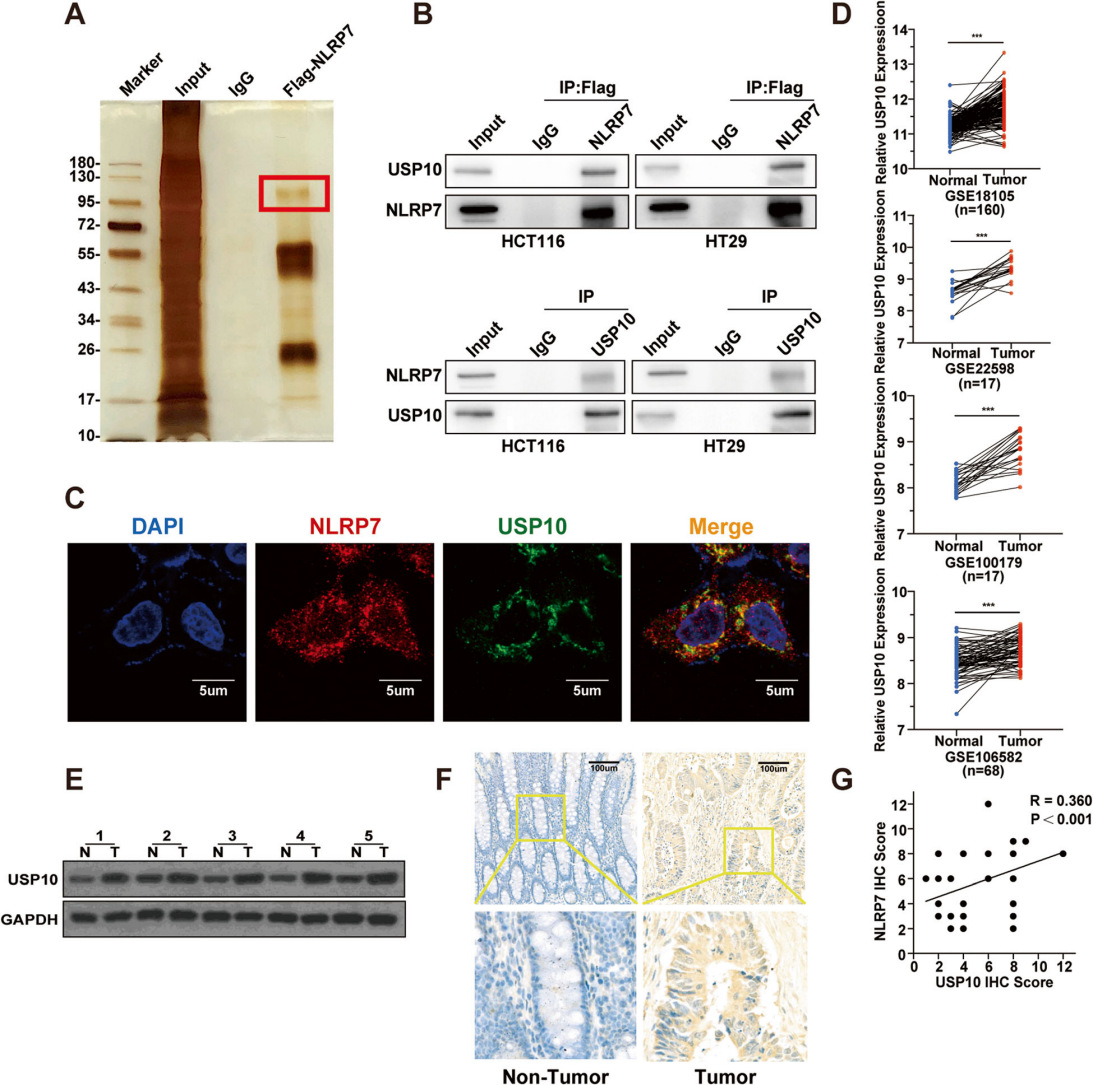
USP10 interacts with NLRP7 and is highly expressed in CRC. **a** Protein pulldown experiment for the identification of proteins associated with NLRP7 by incubation of Flag-NLRP7-antibody with protein extracts from CRC cells. The red box indicates one of the most abundant bands as compared with IgG. **b** IP assay showed that Flag-NLRP7-antibody could pull down USP10 (upper). Correspondingly, USP10-antibody also pulled down NLRP7 (lower). **c** Immunofluorescence showed colocalization of NLRP7 (red) and USP10 (green). Nuclei were stained with DAPI (blue). Scale bars, 5 μm. **d** Analysis of *USP10* gene expression in CRC from the GEO dataset. **e** The protein levels of USP10 in CRC tissues and paired normal tissues measured by WB. N: non-tumor, T: tumor. **f** The USP10 protein in CRC samples and corresponding adjacent normal tissues confirmed by IHC staining. Scale bars, 100 μm. **g** Correlation analysis of NLRP7 and USP10 protein expression in CRC tissues. ***P < 0.001

To identify candidate proteins that interact with NLRP7, a protein pull-down assay was performed. One of the most abundant and specific bands was separated and identified as ubiquitin-specific peptidase 10 (USP10) by liquid chromatography-mass spectrometry (LC/MS-MS) analysis (Fig. 2a), and all proteins that may physically interact with NLRP7 are listed in Additional file 4. IP assays confirmed that USP10 existed in complexes precipitated with antibody against Flag-NLRP7 compared with control IgG (Fig. 2b, upper). Binding of endogenous USP10 to NLRP7 was validated by IP assay with antibody against USP10 (Fig. 2b, lower). IF staining showed that NLRP7 extensively colocalized with USP10 in the cytoplasm (Fig. 2c).

To evaluate the expression profile of USP10 in CRC, we analyzed the GEO database (GSE18105, GSE22598, GSE100179, GSE106582), and the results showed that USP10 mRNA was significantly upregulated in CRC (Fig. 2d). USP10 protein levels were increased in representative CRC patient tissues compared with normal tissues (Fig. 2e). To investigate the function of USP10 in CRC, we performed IHC staining for USP10 using archived CRC tissues as described previously. The results showed that the expression of USP10 was elevated and significantly positively correlated with NLRP7 expression in CRC tissues (Fig. 2f-g).

### Degradation of NLRP7 is repressed by USP10 deubiquitination

**Fig. 3:**
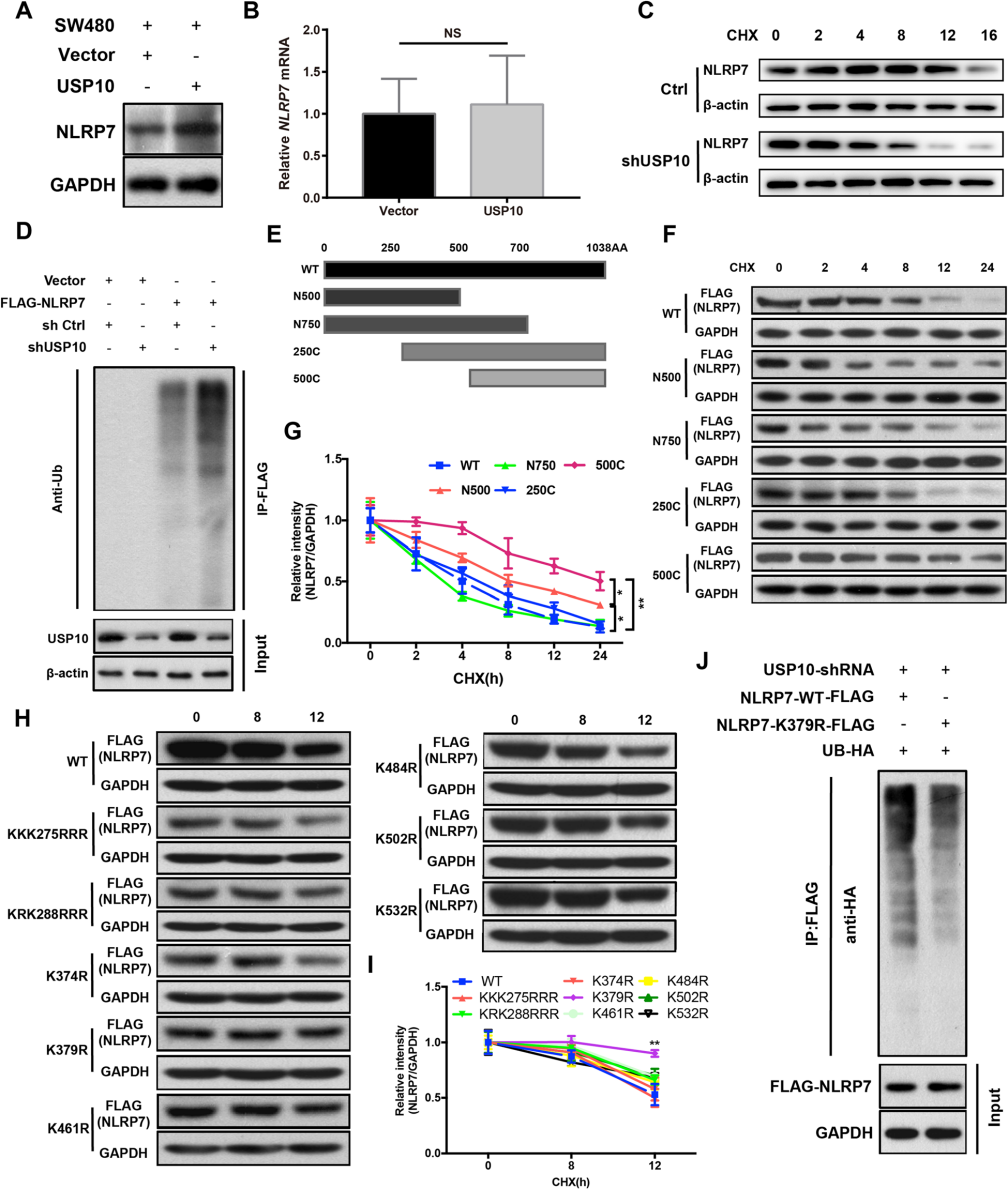
Degradation of NLRP7 is repressed by USP10 deubiquitination. **a** USP10 upregulation increased NLRP7 protein levels, but (**b**) had no significant effect on mRNA levels of NLRP7 in CRC cells. **c** In cycloheximide (CHX) chase, NLRP7 protein stability was decreased in HCT116 cells with knockdown of USP10 compared to control. **d** USP10 knockdown increased the ubiquitination of NLRP7 in HCT116 cells. **e** Mapping of NLRP7 using truncation mutants. **f** Half-life analysis of transfected wild-type and truncation mutant NLRP7-FLAG and (**h**) transfected K-R point mutant NLRP7-FLAG in CRC cells. Densitometric analysis of (**g**) NLRP7 truncation mutant and (**i**) NLRP7 K-R point mutant signal versus time. **j** Immunopurified NLRP7-FLAG K379 mutant showed decreased ubiquitination compared to wild-type in HCT116 cells with shUSP10. **P < 0.01, and *P < 0.05. NS: no significance

To confirm the positive role of USP10 in the regulation of NLRP7, we investigated the effect of changes in USP10 expression on NLRP7 in HCT116 cells. USP10 upregulation increased NLRP7 protein levels (Fig. 3a). However, USP10 had no significant effect on the mRNA levels of NLRP7 in CRC cells (Fig. 3b). Considering that USP10 is a deubiquitinase that removes ubiquitin from its substrates [28], we analyzed the effects of USP10 on the deubiquitination and degradation of NLRP7. Consistently, knockdown of USP10 in HCT116 cells significantly shortened the half-life of the NLRP7 protein in the presence of the protein synthesis inhibitor cycloheximide (CHX) compared with that under control conditions (Fig. 3c). These results suggest that NLRP7 levels are modulated post-translationally through its degradation. Co-IP experiments showed that USP10 knockdown increased the ubiquitination of NLRP7 in HCT116 cells (Fig. 3d).

Next, we identified the lysine on NLRP7 that binds to the Ub moiety in CRC cells. Human NLRP7 truncation mutants were cloned into the hU6-MCS-CBh-gcGFP-IRES-puromycin vector, and the protein stability of ectopically expressed mutants was assessed in the presence of the protein synthesis inhibitor CHX. A significant increase in protein stability was only observed in the 500C NLRP7 mutant (Fig. 3e-g), suggesting that the NLRP7 ubiquitin acceptor site resides within 10 lysine residues between aa 250 and 500. Point mutations were then generated by substituting arginine for lysine at each candidate acceptor site. The protein stability of the K379R mutant increased significantly in cells treated with CHX (Fig. 3h-i). Co-IP studies confirmed that ubiquitination was decreased in K379R NLRP7 compared with that in wild-type NLRP7 (Fig. 3j). These results suggest that K379 is an important lysine acceptor site involved in modulating NLRP7 half-life in CRC cells.

### USP10 accelerates the CRC malignant process by upregulating NLRP7

**Fig. 4:**
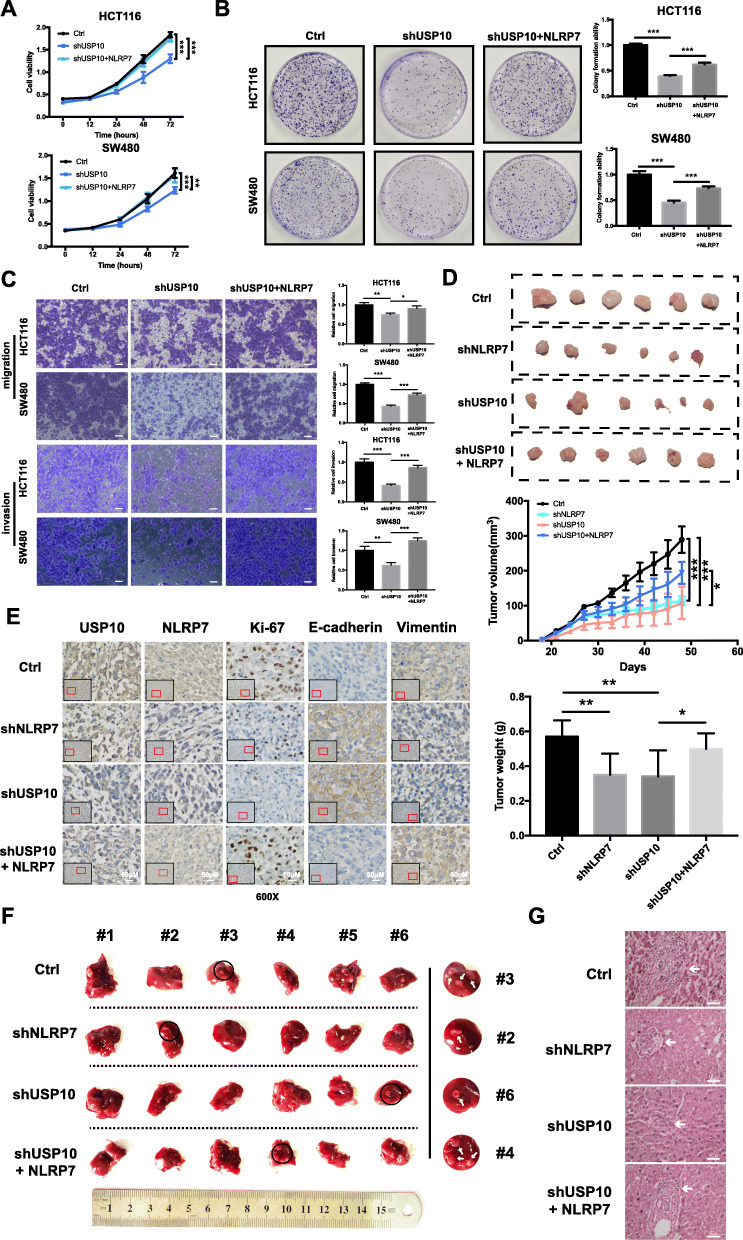
USP10 accelerates the CRC malignant process by upregulating NLRP7. **a-c** NLRP7 overexpression rescued impaired proliferation, colony formation, migration, and invasive capability caused by USP10 knockdown in HCT116 and SW480 cells. Scale bars, 100 μm. **d** NLRP7 or USP10 knockdown inhibited the growth of HCT116 tumors in vivo, whereas overexpression of NLRP7 in USP10 knockdown cells had a synergistic effect on tumor growth. **e** Representative IHC images of Ki-67, E-cadherin, and vimentin in different xenograft sections (magnification: × 600). Scale bars, 50 μm. **f** Visible metastatic lesions in the liver, and NLRP7 overexpression reversed the inhibition of liver metastasis by USP10 knockdown. **g** Representative images of hematoxylin-eosin staining sections from metastatic nodules in the liver (magnification: × 200). Scale bars, 25 μm. ***P < 0.001, **P < 0.01, and *P < 0.05

To explore the role of NLRP7 in the tumor-promoting function of USP10, the effect of NLRP7 overexpression on the tumorigenic potential of USP10-shRNA lentivirus-stable–transfected HCT116 and SW480 cells was examined. Downregulation of USP10 expression significantly suppressed the proliferative, migration, and invasive capabilities of HCT116 and SW480 cells, whereas overexpression of NLRP7 attenuated the inhibitory effect of USP10 silencing (Fig. 4a-c).

Tumor xenograft experiments showed that NLRP7 or USP10 knockdown inhibited the growth of HCT116 tumors in vivo, whereas overexpression of NLRP7 in USP10 knockdown cells had a synergistic effect on tumor growth (Fig. 4d). IHC staining for Ki-67, E-cadherin, and vimentin in xenograft tumors was performed and signals were captured under a microscope (Fig. 4e). NLRP7 or USP10 knockdown dramatically inhibited liver metastasis in a mouse model. However, NLRP7 overexpression reversed the inhibition of liver metastasis by USP10 knockdown (Fig. 4f-g). Collectively, the in vitro and in vivo results suggest that NLRP7 is involved in the effect of USP10 on promoting tumor progression and metastasis in CRC.

### NLRP7 promotes CCL2 expression and TAM polarization

**Fig. 5:**
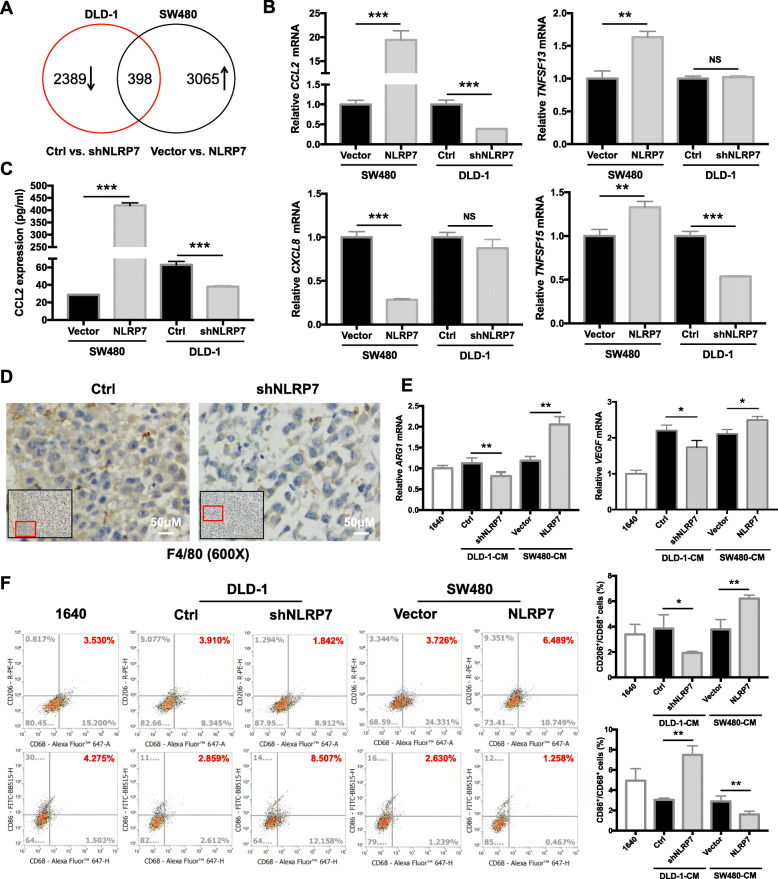
NLRP7 promotes CCL2 expression and TAM polarization. **a** Number of altered transcripts detected in CRC cells with either NLRP7 overexpression or knockdown. **b** qRT-PCR verification of transcription of four cytokines/chemokines, including CCL2, TNFSF13, CXCL8, and TNFSF15, in CRC cells with NLRP7 overexpression or knockdown. **c** ELISA confirmed CCL2 secretion levels in CRC cells with NLRP7 overexpression or knockdown. **d** Macrophages detected by immunohistostaining of the xenograft tumor tissues from NLRP7 knockdown and control groups (magnification: × 600). Scale bars, 50 μm. **e** Expression levels of M2-like macrophage marker changes after treatment with culture medium from NLRP7 overexpressing or knockdown CRC cells. **f** Flow cytometry was used to evaluate CD68(+)/CD206(+) and CD68(+)/CD86(+) subpopulations. ***P < 0.001, **P < 0.01, and *P < 0.05. NS: no significance

To elucidate the molecular mechanism by which NLRP7 promotes CRC progression, CRC cells with stable NLRP7 overexpression and knockdown were subjected to RNA sequencing (RNA-seq). RNA-seq identified 3065 transcripts that were significantly upregulated by NLRP7 overexpression (fold change > 2) and 2389 transcripts that were significantly downregulated (fold change < 0.5) by NLRP7 knockdown (Fig. 5a). The results showed that 398 genes were positively correlated with NLRP7 expression and overlapped between the two groups. The 398 transcripts are listed in Additional file 5, and the GO and KEGG enrichment analysis results are shown in Additional file 3: Fig. S2A and S2B. Cytokines and chemokines, as downstream signal transduction molecules, are essential for NLR family members to ensure their function and influence disease outcomes [6, 10, 29, 30]. Therefore, we decided to focus on cytokines and chemokines that are regulated by NLRP7 in cancer cells. Among 398 transcripts, four cytokines/chemokines, including CCL2, TNFSF13, CXCL8, and TNFSF15, were positively correlated with the expression of NLRP7. Among them, CCL2 mRNA detected by qRT-PCR was dramatically increased in NLRP7-overexpressing cells and decreased in NLRP7 knockdown cells (Fig. 5b). ELISA with a specific anti-CCL2 antibody confirmed that CCL2 secretion was induced by NLRP7 overexpression and significantly repressed by NLRP7 knockdown (Fig. 5c). Because high levels of CCL2 are correlated with increased numbers of TAMs in tumor tissues in many human tumors [31], we speculated that the recruitment of TAMs by CCL2 is involved in the function of NLRP7 in promoting CRC progression.

To test the hypothesis that NLRP7 regulates the release of chemokines by CRC cells to modulate TAM polarization, THP-1 cells were incubated with conditioned medium (CM) collected from different CRC cell lines, and the expression of TAM polarization markers was analyzed. The culture medium from NLRP7 overexpressing cells promoted the chemotaxis of THP-1 human monocytes (Additional file 3: Fig. S3A). Moreover, macrophages (F4/80) were reduced in the tumor microenvironment (TME) of xenograft tumor tissues in NLRP7 knockdown group compared with control, as detected by immunohistostaining (Fig. 5d). The CM from NLRP7 overexpressing CRC cells, but not the control medium, upregulated the mRNA expression of *VEGF* and *ARG1* which were used as indicators for the functional polarization of TAMs promoted by tumor signals [32] (Fig. 5e). Flow cytometry showed that the CD68(+)/CD206(+) subpopulations were more abundant, whereas CD68(+)/CD86(+) subpopulations were less abundant in cells overexpressing NLRP7 than in the controls (Fig. 5f). The opposite effect was observed in THP-1 cells incubated with CM from NLRP7 knockdown cells.

### CCL2 secretion from CRC cells is necessary for NLRP7 inducing TAM polarization

**Fig. 6:**
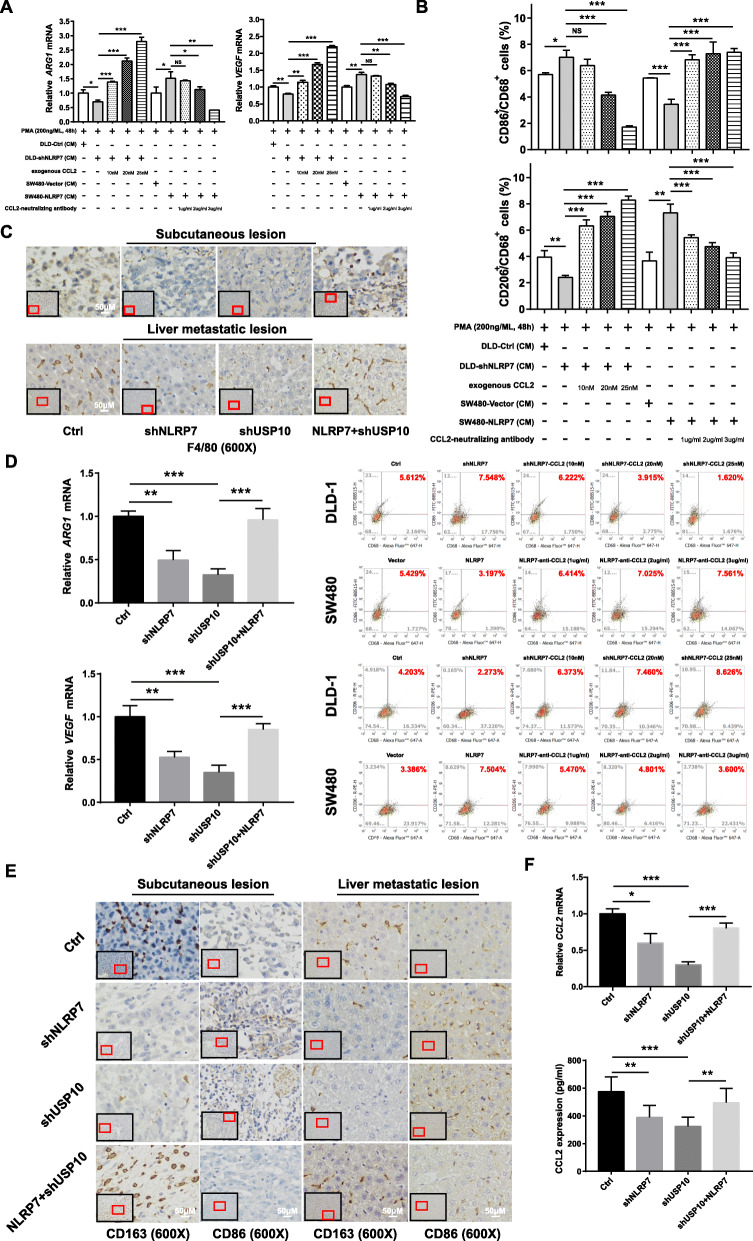
CCL2 secretion from CRC cells is necessary for NLRP7 induction of TAM polarization. **a-b** The results of qPCR and flow cytometry confirmed that recombinant CCL2 rescued the M2-like macrophage polarization effect from CM of NLRP7 knockdown cells, and CCL2-neutralizing antibody suppressed M2-like macrophage polarization effect from CM of NLRP7 overexpressing cells. **c** Macrophages in xenograft tumor tissues from control, NLRP7 knockdown, USP10 knockdown, and USP10 knockdown with NLRP7 overexpression (magnification: × 600). Scale bars, 50 μm. **d** Expression level of TAM functional polarization markers in xenograft tumor tissues. **e** CD86-positive M1 macrophages and CD163-positive M2 macrophages in xenograft tumor tissues from four groups (magnification: × 600). Scale bars, 50 μm. **f** The expression levels of CCL2 in peripheral blood and tumor tissues of animals from four groups. ***P < 0.001, **P < 0.01, and *P < 0.05. NS: no significance

To investigate whether the effect of CRC cells on TAM polarization is mediated by the secretion of CCL2, recombinant CCL2 and a CCL2-neutralizing antibody were added to the CM from NLRP7 knockdown cells and the CM from NLRP7 overexpressing cells, respectively. The results of qPCR and flow cytometry confirmed that CCL2 induced M2-like macrophage polarization in a dose-dependent manner (Fig. 6a-b). Moreover, the CM from SW480 cells treated with CCL2-neutralizing antibody significantly inhibited the chemotaxis of THP-1 cells, which was restored by exogenous CCL2 addition (Additional file 3: Fig. S3B).

In animal studies, macrophages were reduced in the TME of xenograft tumor tissues in NLRP7 and USP10 knockdown groups and were increased in the NLRP7 overexpressing group (Fig. 6c). The Vegf and Arg1 mRNA expression in tissues was higher in NLRP7-overexpressing tumors and lower in sh-NLRP7 and sh-USP10 tumors than in the control group (Fig. 6d). Furthermore, IHC staining for the M1 marker CD86 showed that accumulation of CD86-positive M1 macrophages was greater in the stroma of sh-NLRP7 tumors and sh-USP10 tumors than in that of scrambled shRNA treated tumors, and NLRP7 overexpression suppressed the accumulation of CD86-positive M1 macrophages. CD163-positive M2 macrophages were more abundant in NLRP7-overexpressing tumors and less abundant in sh-NLRP7 tumors and sh-USP10 tumors than in the control tumors (Fig. 6e). The expression levels of CCL2 in peripheral blood and tumor tissues were significantly lower in sh-NLRP7-treated and sh-USP10-treated mice than in control mice and higher in NLRP7-treated mice with USP10 knockdown (Fig. 6f). Thus, data from cells and animal models suggested that NLRP7 enhances TAM polarization by promoting CCL2 secretion from CRC cells.

### NLRP7 facilitates transactivation of CCL2 via NF-κB

**Fig. 7:**
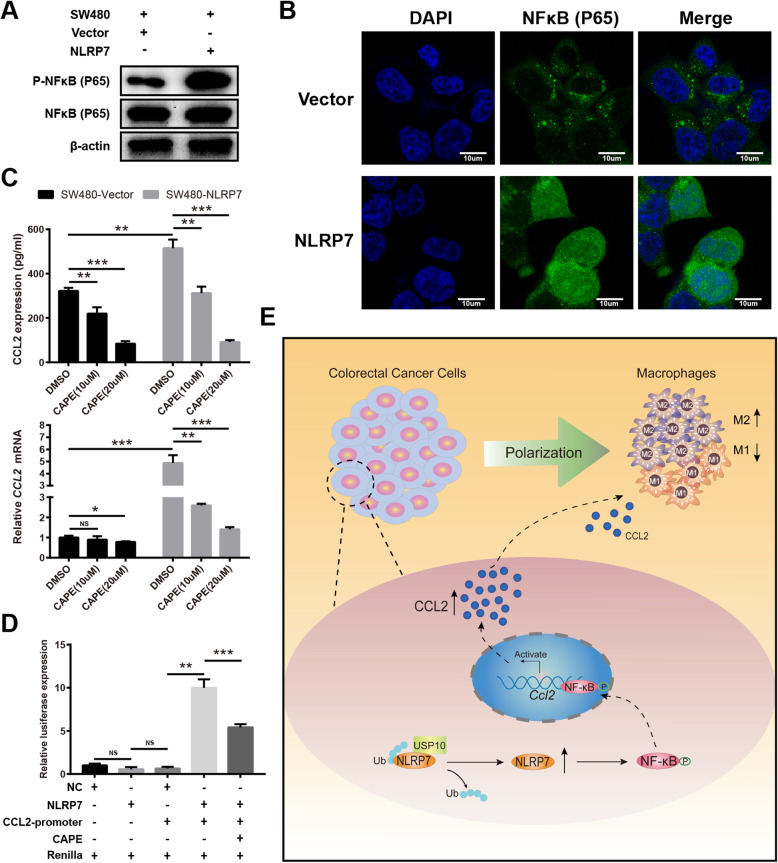
NLRP7 facilitates transactivation of CCL2 via NF-κB. **a** Phosphorylation of p65 was increased in NLRP7-overexpressing cells, whereas the protein levels did not change. **b** Immunofluorescence analysis demonstrated that NLRP7 overexpression increased p65 NF-κB accumulation in the nucleus. Scale bars, 10 μm. **c** NF-κB-specific inhibitor CAPE suppressed the NLRP7-induced expression of CCL2. **d** Luciferase reporter gene analysis showed that ectopic expression of NLRP7 induced, whereas CAPE suppressed CCL2 transcription. **e** A schematic diagram displays high level of NLRP7 protein deubiquitination by USP10 promoting tumor-associated macrophage polarization via NF-κB-CCL2 signaling in colorectal cancer. ***P < 0.001, **P < 0.01, and *P < 0.05. NS: no significance

Considering that CCL2 is a classical NF-κB target gene [25], we next examined whether the effect of NLRP7 on CCL2 expression and monocyte chemotaxis is mediated by enhanced NF-κB signaling. The NF-κB circuit involves p65 phosphorylation and nuclear translocation, which lead to gene activation. Phosphorylation of p65 was increased in NLRP7-overexpressing cells, whereas the protein levels did not change (Fig. 7a). IF analysis demonstrated that NLRP7 overexpression increased p65 NF-κB accumulation in the nucleus compared with that in control cells (Fig. 7b). These results suggest that NLRP7 promotes p65 NF-κB accumulation in the nucleus by enhancing its phosphorylation.

Next, we determined the effect of NLRP7 on CCL2 expression in response to NF-κB activation. We confirmed that the NLRP7-induced expression of CCL2 was significantly suppressed following NF-κB inhibition using the NF-κB-specific inhibitor CAPE (Fig. 7c). Luciferase reporter gene analysis showed that ectopic expression of NLRP7 induced, whereas CAPE suppressed CCL2 transcription (Fig. 7d). These results suggest that NLRP7 regulates the expression of CCL2 by modulating the NF-κB signaling pathway.

Collectively, the present results indicated that NLRP7 in CRC cells interacts with and is deubiquitinated by USP10, resulting in increased NLRP7 protein stability (Fig. 7e). NLRP7 promoted the secretion of CCL2 by activating the NF-κB signaling pathway. CCL2 secreted by CRC cells promoted the polarization of pro-tumor M2-like macrophages. Therefore, upregulation of NLRP7 in CRC facilitated tumor cell proliferation and metastasis.

## Discussion

NLRs affect multiple stages of cancer progression in several malignancies, including colorectal cancer, gastric cancer, hepatocellular carcinoma, and other cancers [6]. The exact roles of NLR family members in CRC are diverse and complex. In this study, we showed that NLRP7 is upregulated at the protein level in CRC samples compared with matched non-tumor controls, and that overexpression of NLRP7 is associated with poor prognosis of CRC patients. Functional studies in vitro and in vivo revealed that NLRP7 promoted tumor cell proliferation and metastasis. Previous studies focused on the role of NLRP7 during embryonic development [33]. The present results thus expand our knowledge of the role of this protein in cancer.

At the beginning of this study, we observed an increase in NLRP7 protein levels without changes in mRNA levels in CRC tissues compared with levels in the corresponding ANTs. This phenomenon needed to be explained mechanistically. Protein ubiquitination is an important post-translational modification that regulates the degradation of proteins, but not alteration of mRNA [34]. A recent study showed that STAM-binding protein (STAMBP), a deubiquitinating enzyme, modulates NLRP7 protein stability by catalyzing its deubiquitination to increase NLRP7 abundance [35]. Moreover, some other NLR family members, including NLRP3 and NOD2, were also demonstrated to be affected by de-ubiquitinase enzymes (DUBs) [36–39]. Even without experimental validation, the above literature suggests that NLRP7 regulated by deubiquitination is a well-founded hypothesis. Here, we identified USP10 as a critical regulator of NLRP7 deubiquitination and stabilization in CRC cells. We identified K379 as an important lysine acceptor site that mediates NLRP7 ubiquitination in CRC cells. However, a previous study identified KRK288/290 as the main lysine acceptor sites affecting ubiquitination of NLRP7 [35]. This discrepancy may be related to differences in the cells and conditions used for analyzing NLRP7, as well as different biological functions of NLRP7.

Several lines of evidence support the diverse roles of USP10 in tumorigenesis in different cancer types. For example, in hepatocellular carcinoma, Zhu et al. reported that USP10 promotes proliferation [40], whereas Lu et al. found that USP10 suppresses tumor progression by inhibiting mTOR activation [41]. In the present study, USP10 was overexpressed in CRC tissues, as determined by our data and public GEO database information. Targeting USP10 by shRNA inhibited CRC proliferation and metastasis, and this effect was reversed by overexpression of NLRP7. This result indicates that NLRP7 may be a key effector molecule for the cancer-promoting effects of USP10 in CRC cells.

TAMs constitute a plastic and heterogeneous cell population of the tumor microenvironment, and they are closely related to cancer progression and resistance to therapy [42]. Here, we found that NLRP7 overexpression in CRC cells enhanced TAM polarization, which may be one of the mechanisms by which NLRP7 promotes CRC progression. Polarization of TAMs is regulated by multiple microenvironmental cytokines, growth factors, epigenetic regulators, and other signals derived from tumor and stromal cells [43]. We showed that NLRP7 promoted the transcription of CCL2, and the secretion of CCL2 was increased in NLRP7 overexpressing CRC cells. Several studies reported that CCL2 not only promotes tumor invasion and metastasis, but also acts as a macrophage recruiter and M2-stimulating factors [44–46]. We showed that M2-like macrophage polarization induced by overexpression of NLRP7 in CRC cells is mediated by CCL2, and this effect can be attenuated by a CCL2-neutralizing antibody in vitro. Moreover, the protein levels of CCL2 in peripheral blood were significantly decreased in sh-NLRP7-treated mice and increased in NLRP7-treated mice, whereas M2-like macrophages were more abundant in NLRP7-overexpressing tumors and less abundant in sh-NLRP7 tumors. These results indicate that high expression of NLRP7 in CRC promotes macrophage polarization in a CCL2-dependent manner.

Mechanistically, we demonstrated that the NF-κB pathway is essential for CCL2 production induced by NLRP7 in CRC cells. We confirmed that NLRP7 promotes the phosphorylation of p65 NF-κB, leading to increased nuclear accumulation of p65 NF-κB. NLRs were previously shown to modulate NF-κB signaling by inhibition or activation [47]. NLRC1 activates NF-κB signaling [48], whereas NLRP12 negatively regulates NF-κB [13]. In this study, the translocation of NF-κB from the cytoplasm to the nucleus promoted by NLRP7 enhanced the binding of NF-κB to the CCL2 promoter, thereby increasing its expression and secretion. Previous studies showed that the genes encoding CCL2 are downstream targets of the NF-κB transcription factor, which are crucial for cancer progression and metastasis [25, 49]. Therefore, the NF-κB-CCL2 signaling axis plays a critical role in the effect of NLRP7 on promoting TAM polarization and CRC progression.

## Conclusion

In summary, we identified a potential role of NLRP7 in the pathogenesis and metastasis of CRC. NLRP7 protein levels were increased by USP10-mediated deubiquitination in CRC cells, which induced the polarization of pro-tumor M2-like macrophages via NF-κB pathway-mediated CCL2 secretion. These findings provide a better understanding of the oncogenic mechanisms of NLRP7, and may advance the development of therapeutic strategies for CRC.

## Supplementary Information

**Additional file 1: Table S1.** Primer sequences for real-time PCR. **Table S2.** The antibodies used in western blot, flow cytometry, and IHC. **Table S3.** Primer sequences for truncation mutant and site-directed mutagenesis. **Table S4.** Clinicopathological correlation of high NLRP7 expression in CRC.

**Additional file 2:** Unprocessed original blots.

**Additional file 3: Figure S1. (A)** Analysis of *NLRP7* gene expression from the GEO dataset. **(B)** Representative images of colony formation assay. **(C)** Cell apoptosis in NLRP7 knockdown and overexpression CRC cells manifested by flow cytometry analysis. **Figure S2.** The GO **(A)** and KEGG **(B)** enrichment analysis results of 398 genes positively related with NLRP7 expression. **Figure S3. (A)** Chemotaxis of THP-1 cells treated with culture medium from cells overexpressing NLRP7**. (B)** CCL2-neutralizing antibody inhibited THP-1 cell chemotaxis, which was restored by exogenous CCL2.

**Additional file 4:** The list of proteins physically interacted with NLRP7.

**Additional file 5:** The list of 398 genes that were positively correlated with NLRP7 expression in CRC cells.

## Data Availability

All data in our study will be available upon reasonable request.

## Abbreviations

CRC: Colorectal cancer
TME: Tumor microenvironment
TAMs: Tumor-associated macrophages
CCL2: C-C motif chemokine 2
NLRs: NOD-like receptors
PAMPs: Pathogen-associated molecular patterns
NLRP7: NACHT, LRR and PYD domains-containing protein 7
UC: Ulcerative colitis
IBD: Inflammatory bowel disease
OS: Overall survival; USP10: ubiquitin-specific-processing protease 10
ANTs: Adjacent normal tissues
IHC: Immunohistochemistry
IP: Immunoprecipitation
IF: Immunofluorescence
CHX: Cycloheximide
CM: Conditioned medium
