# Supplementary material for: NLRP7 deubiquitination by USP10 promotes tumor progression and tumor-associated macrophage polarization in colorectal cancer
Source: J Exp Clin Cancer Res. 2021 Apr 10;40:126. doi: 10.1186/s13046-021-01920-y (PMC8035766; doi:10.1186/s13046-021-01920-y)
Supplement: Supplementary file 1 — Additional file 1: Table S1. Primer sequences for real-time PCR. Table S2. The antibodies used in western blot, flow cytometry, and IHC. Table S3. Primer sequences for truncation mutant and site-directed mutagenesis. Table S4. Clinicopathological correlation of high NLRP7 expression in CRC. [file 13046_2021_1920_MOESM1_ESM.docx]

| **Table S1** | | |
| --- | --- | --- |
| **qRT-PCR primers** | | |
| Target | Forward | Reverse |
| NLRP7 | CTG CAG ATG TTG TCG TTG G | CTT GCT GAC AAC CAA GAC AG |
| CCL2 | CAGCCAGATGCAATCAATGCC | TGGAATCCTGAACCCACTTCT |
| TNFSF13 | GGCAACCAGCTCTTAGGCG | AAGTCACGTCTTGAAACAGGAC |
| CXCL8 | ACTGAGAGTGATTGAGAGTGGAC | AACCCTCTGCACCCAGTTTTC |
| TNFSF15 | TACTCCCAGGTCACATTCCGT | GGGTAGCTGTCTGTTACCTTGG |
| H-ARG1 | TGGACAGACTAGGAATTGGCA | CCAGTCCGTCAACATCAAAACT |
| m-ARG1 | CTCCAAGCCAAAGTCCTTAGAG | AGGAGCTGTCATTAGGGACATC |
| H-VEGF | AGGGCAGAATCATCACGAAGT | AGGGTCTCGATTGGATGGCA |
| m-VEGF | GCACATAGAGAGAATGAGCTTCC | CTCCGCTCTGAACAAGGCT |
| H-GAPDH | GGAGCGAGATCCCTCCAAAAT | GGCTGTTGTCATACTTCTCATGG |
| m-GAPDH | AGGTCGGTGTGAACGGATTTG | GGGGTCGTTGATGGCAACA |

| **Table S2** | |
| --- | --- |
| **Antibody** | **Company** |
| Beta-actin | Abcam |
| GAPDH | Cell Signaling Technology |
| NLRP7 | Abcam |
| USP10 | Abcam |
| FLAG | Cell Signaling Technology |
| Ubiquitin | Abcam |
| HA tag | Cell Signaling Technology |
| Ki-67 | Abcam |
| E-cadherin | Abcam |
| Vimentin | Abcam |
| CD86 (for flow cytometry ) | BD Biosciences |
| CD86 (for IHC ) | Abcam |
| CD206 (for flow cytometry ) | BD Biosciences |
| CD68 (for flow cytometry ) | BD Biosciences |
| F4/80( for IHC ) | Abcam |
| CD163 (for IHC ) | Abcam |
| P65 | Abcam |
| phosphor-P65 | Abcam |

| **Table S3** | | |
| --- | --- | --- |
| **Truncation mutant primers** | | |
| WT NLRP7 (forward) | CAC CAT GTC TGA CCA TGG AGA TGT GAG | |
| WT NLRP7 (reverse) | GCA AAA AAA GTC ACA GCA CGG AGG | |
| N500 (reverse) | TAC GTC CCC GAT GTC CCA GG | |
| N750 (reverse) | CAG CAT CAT CGT GCG TTC CCA | |
| 250C (forward) | CAC CAT GGT GGT CGA TGG CC | |
| 500C (forward) | CAC CAT GCA GAA GCT GCT TTC CG | |
| **Site-directed mutagenesis primers** | | |
| Target | Forward | Reverse |
| KKK275RRR | GCG GGG ACT GGG AGA GAA GAA GAC CGG TGC CCG TCC TC | GAG GAC GGG CAC CGG TCT TCT TCT CTC CCA GTC CCC GC |
| KRK288RRR | GGG GAG TTT GCT GAG AAG ACG GAT GTT ACC CAG GG | CCC TGG GTA ACA TCC GTC TTC TCA GCA AAC TCC CC |
| K374R | GTG CAC GAC TCT GAG ACT GCA GAT GGA GAA G | CTT CTC CAT CTG CAG TCT CAG AGT CGT GCA C |
| K379R | GCA GAT GGA GAG AGG GGA GGA CCC G | CGG GTC CTC CCC TCT CTC CAT CTG C |
| K461R | GAC AGA GTC TCC AGA GGC TGC TAC TC | GAG TAG CAG CCT CTG GAG ACT CTG TC |
| K484R | CTA CGC CCT GGA GAG AGA GGA GGG GGA GG | CCT CCC CCT CCT CTC TCT CCA GGG CGT AG |
| K502R | GGG ACG TAC AGA GAC TGC TTT CCG G | CCG GAA AGC AGT CTC TGT ACG TCC C |
| K532R | GAG AAG AGA GCC AGA GAG TTG GAG GCC AC | GTG GCC TCC AAC TCT CTG GCT CTC TTC TC |

| **Table S4. Clinicopathological correlation of NLRP7 High in CRC** | | | | |
| --- | --- | --- | --- | --- |
| **Clinical features** | **Cases** | **NLRP7 expression** | | **P value** |
|  |  | **Low group**  **n. (%)** | **High group**  **n. (%)** |  |
| Age (years old) |  |  |  | 0.670 |
| ≤59 | 63 | 23 (36.5%) | 40 (63.5%) |  |
| >59 | 52 | 21 (40.1%) | 31 (59.6%) |  |
| Sex |  |  |  | 0.646 |
| Male | 71 | 26 (36.6%) | 45 (63.4%) |  |
| Female | 44 | 18 (40.9%) | 26 (59.1%) |  |
| Location |  |  |  | 0.729 |
| Right | 37 | 15(40.5%) | 22 (59.5%) |  |
| Left | 78 | 29 (37.2%) | 49 (62.8%) |  |
| LN metastasis |  |  |  | 0.400 |
| N0 | 57 | 24 (42.1%) | 33 (57.9%) |  |
| N1 | 58 | 20 (34.5%) | 38 (65.6%) |  |
| Distant metastasis |  |  |  | 0.004 |
| M0 | 64 | 32 (50.0%) | 32 (50.0%) |  |
| M1 | 51 | 12 (23.5%) | 39 (76.5%) |  |
| Clinical stage |  |  |  | 0.033 |
| Early (I−II) | 41 | 21 (51.2%) | 20 (48.8%) |  |
| Advanced (III−IV) | 74 | 23 (31.1%) | 51 (68.9%) |  |
