# Supplementary material for: NLRP7 deubiquitination by USP10 promotes tumor progression and tumor-associated macrophage polarization in colorectal cancer
Source: J Exp Clin Cancer Res. 2021 Apr 10;40:126. doi: 10.1186/s13046-021-01920-y (PMC8035766; doi:10.1186/s13046-021-01920-y)
Supplement: Supplementary file 2 — Additional file 2: Unprocessed original blots. [file 13046_2021_1920_MOESM2_ESM.pdf]

Figure 1B

1st

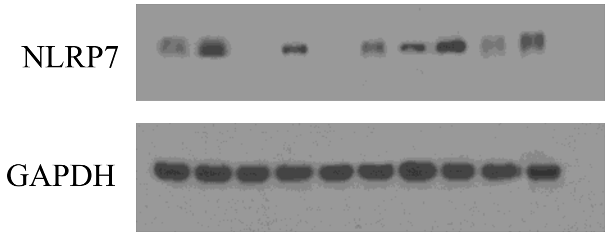

2nd

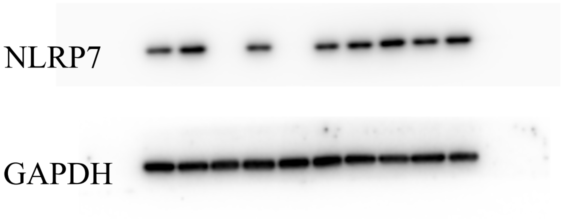

3rd

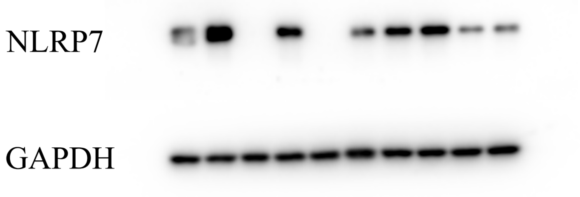

Figure 2B

1st

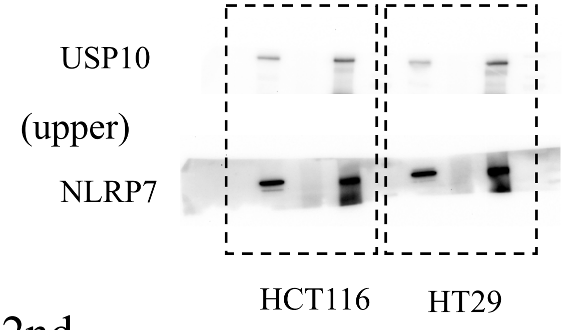

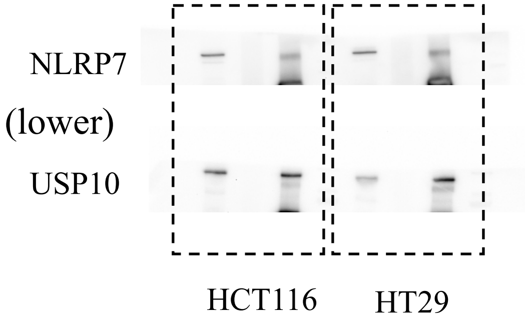

2nd

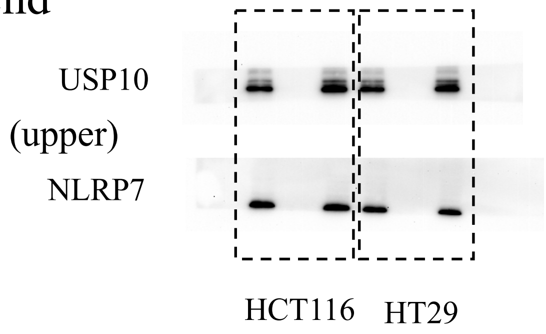

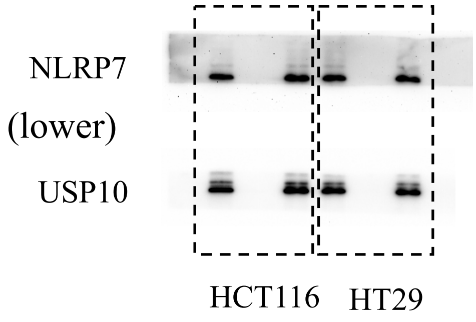

Figure 2B

3rd

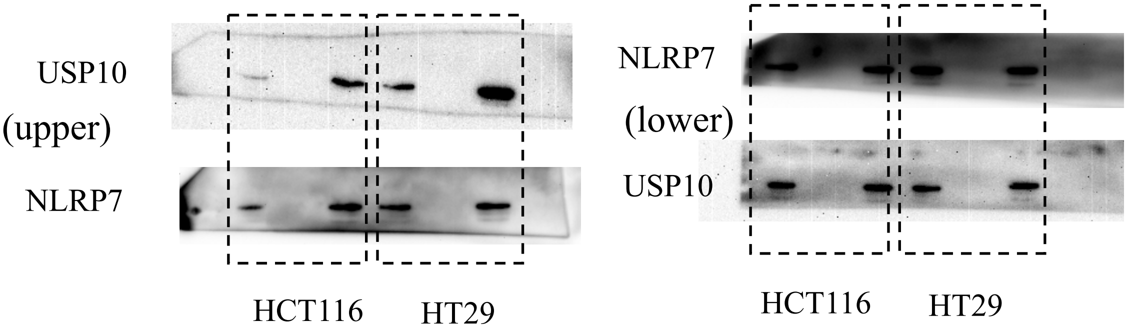

Figure 2E

1st

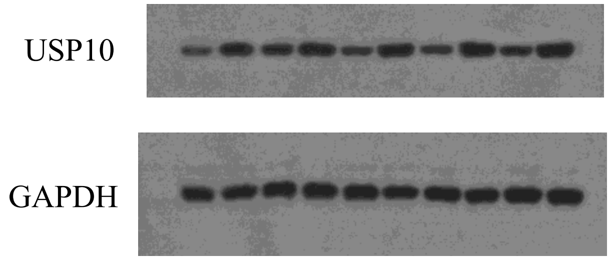

2nd

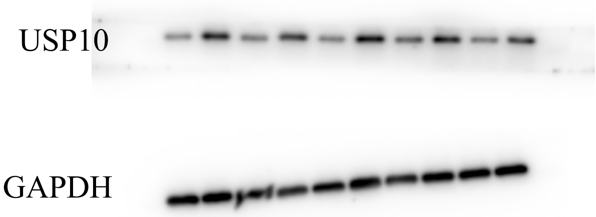

3rd

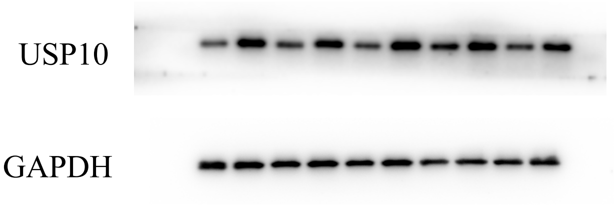

Figure 3A

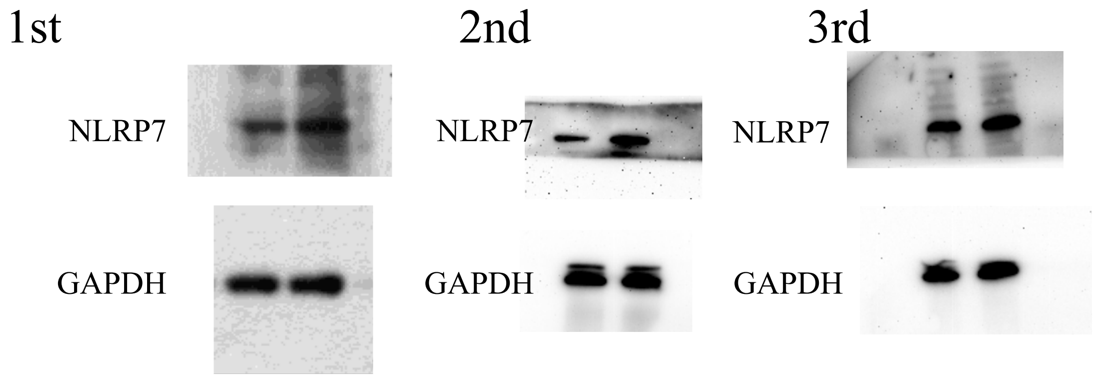

Figure 3C

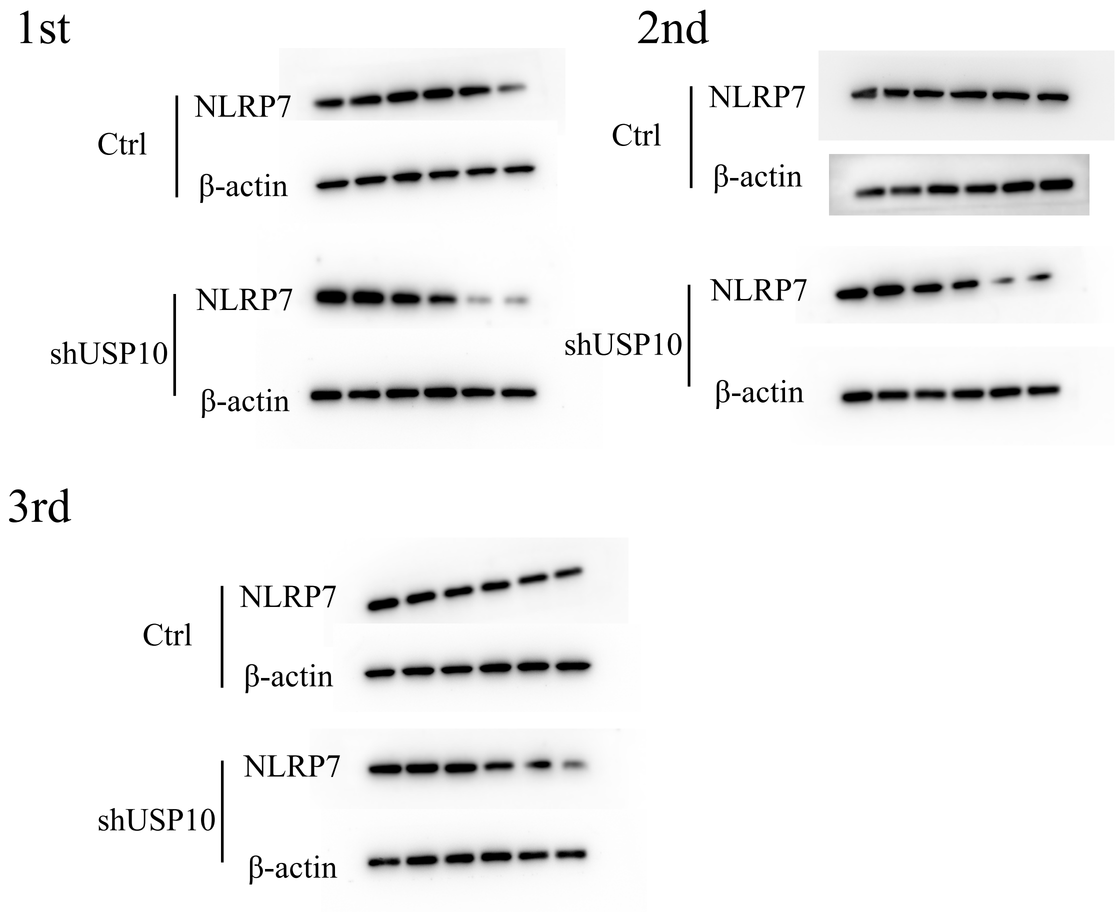

Figure 3D

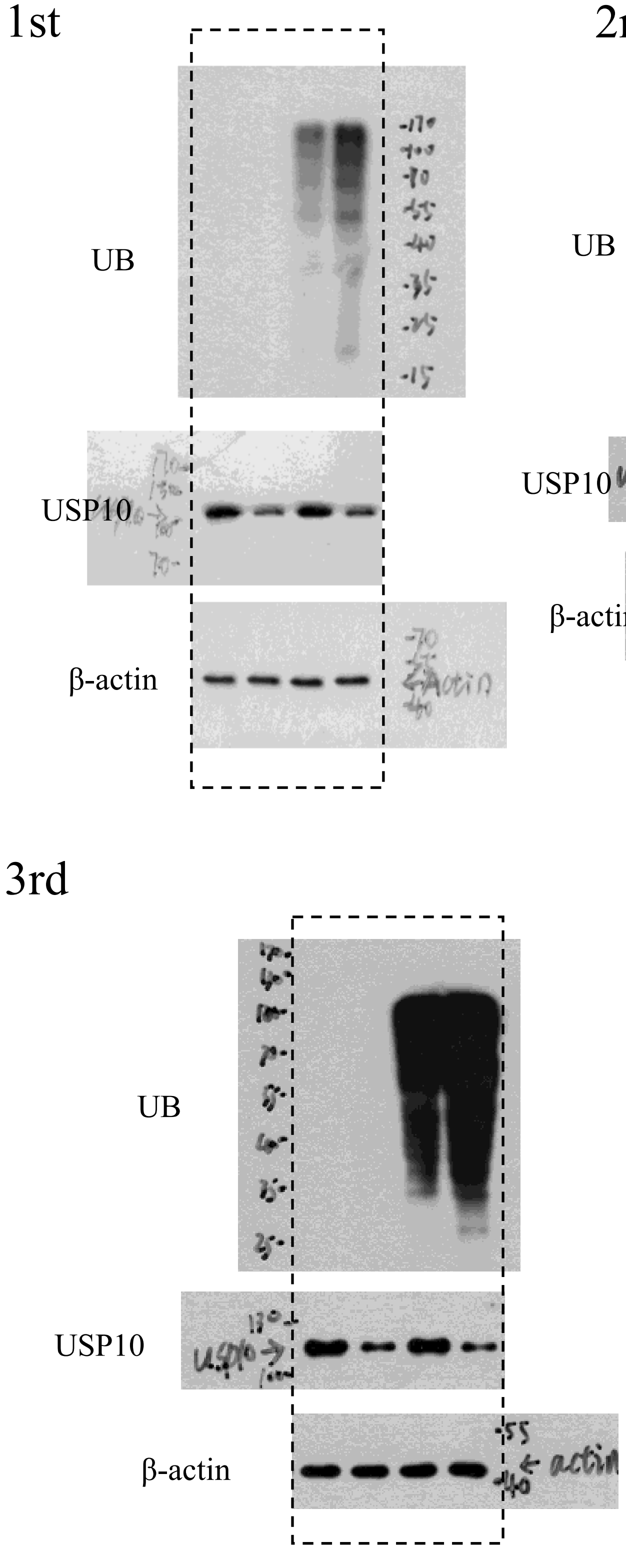

Figure 3F

1st

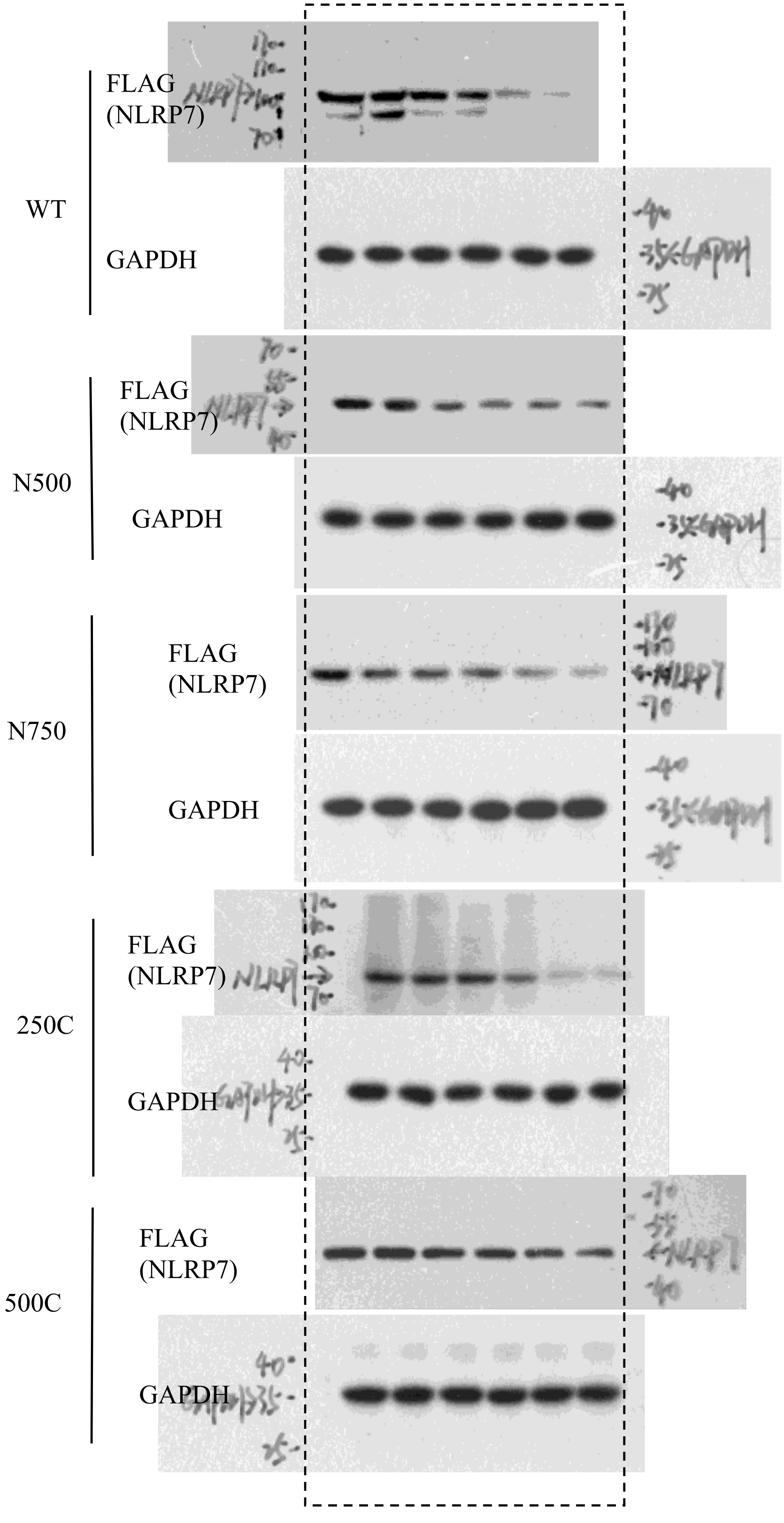

Figure 3F

2nd

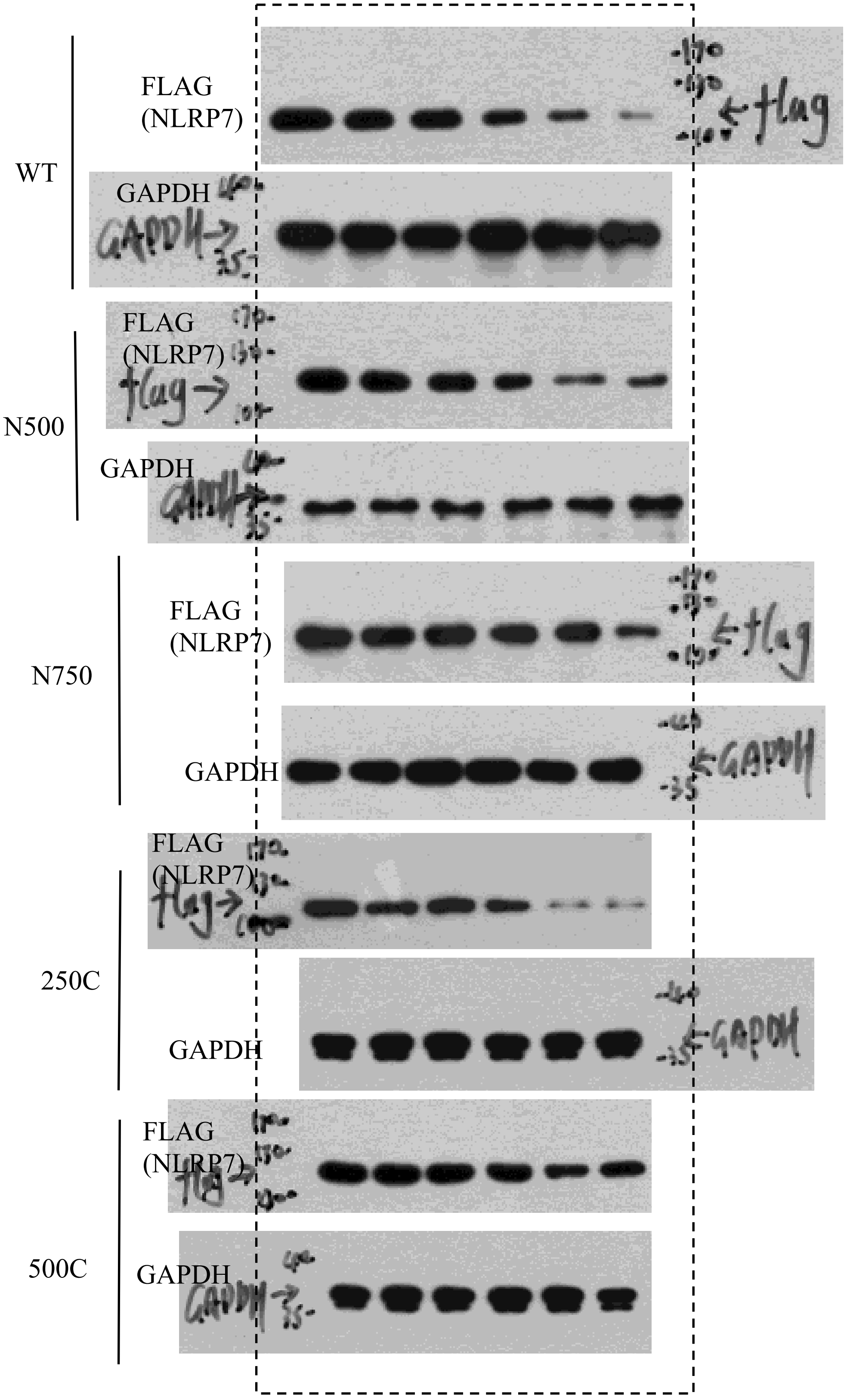

Figure 3F

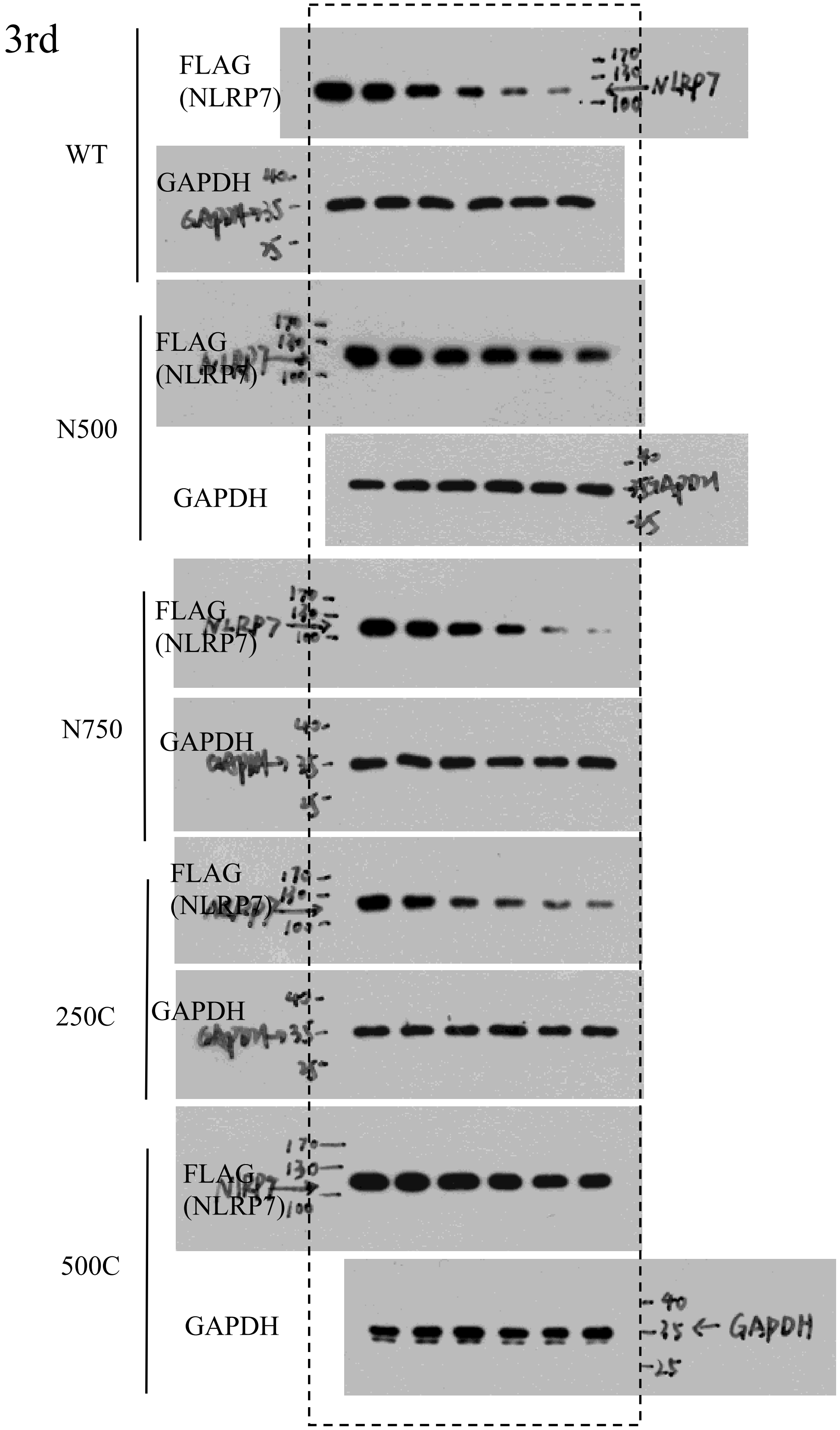
3rd
WT
FLAG (NLRP7)
GAPDH
N500
FLAG (NLRP7)
GAPDH
N750
FLAG (NLRP7)
GAPDH
250C
FLAG (NLRP7)
GAPDH
500C
FLAG (NLRP7)
GAPDH

Figure 3H

1st

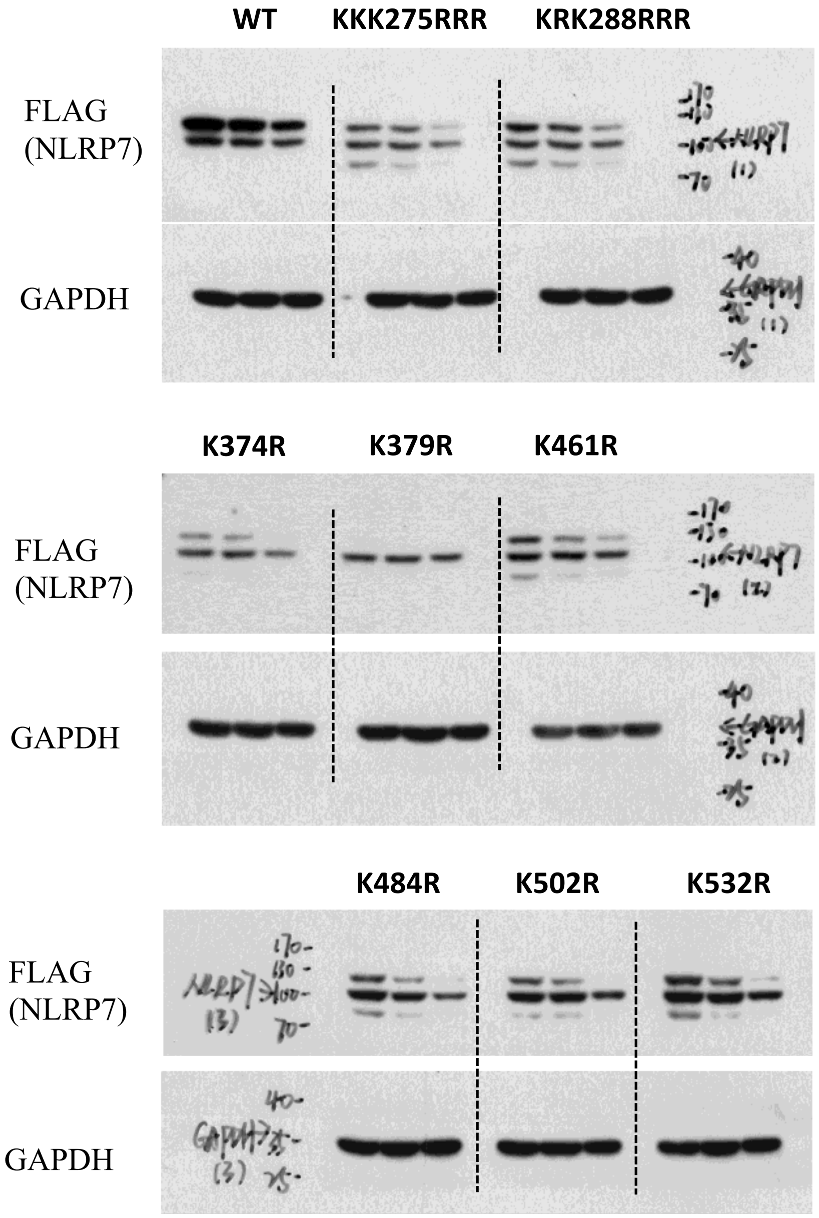

Figure 3H

2nd

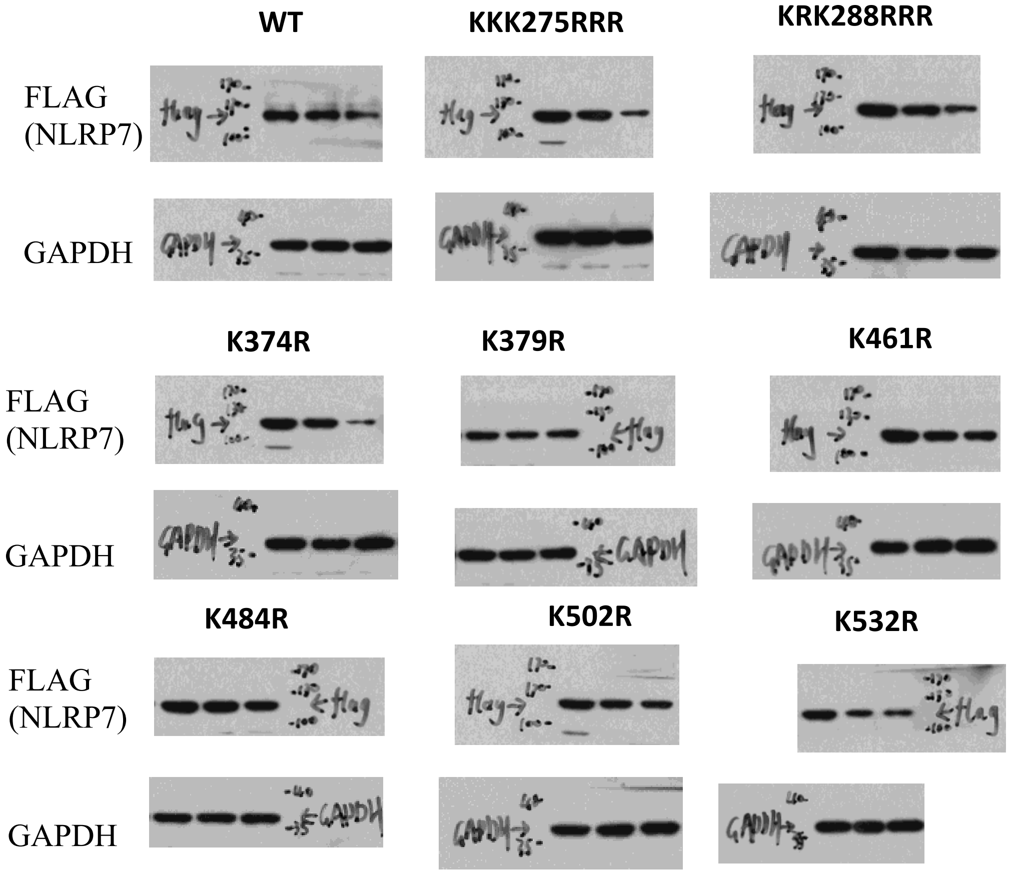

Figure 3H

3rd

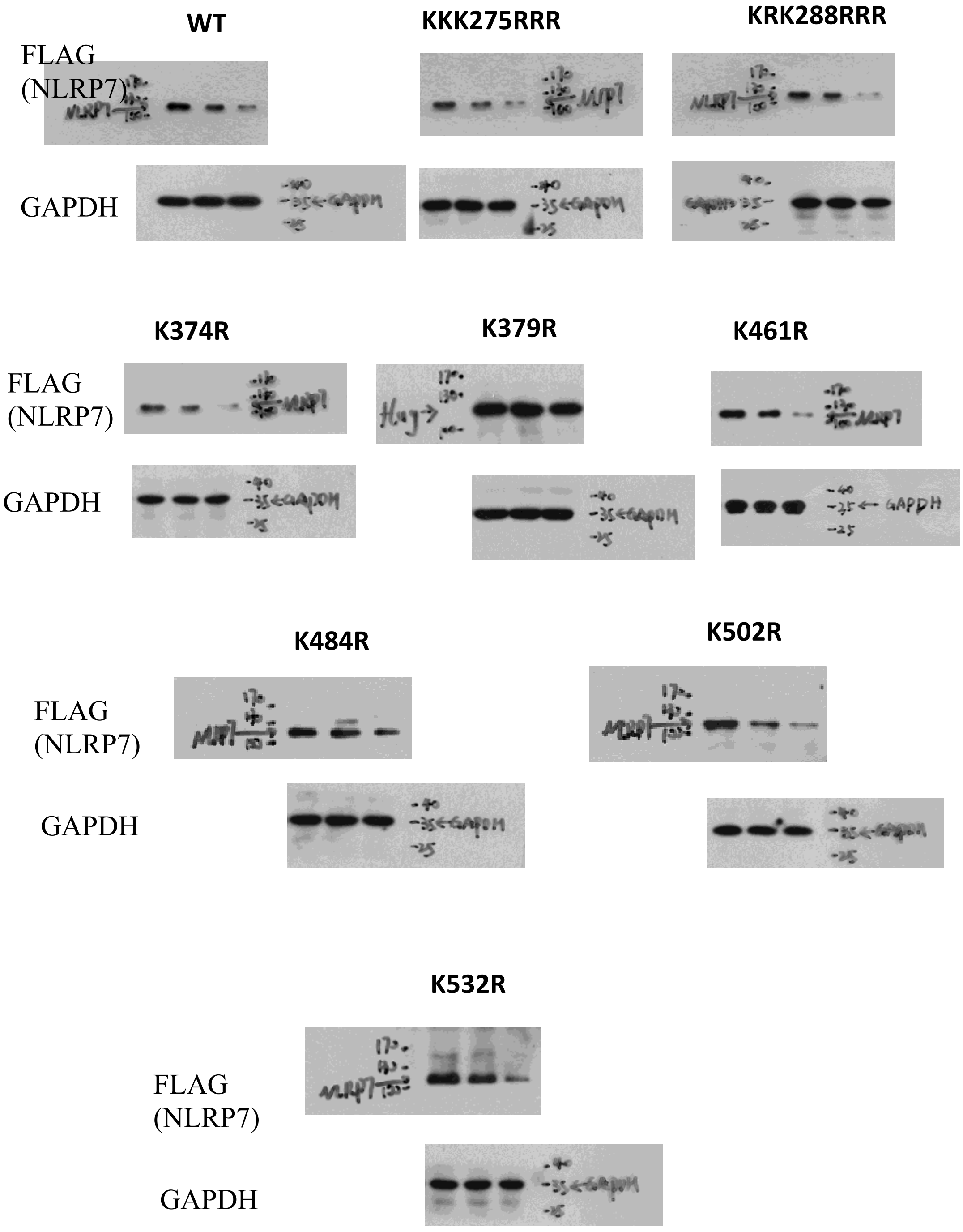

Figure 3J

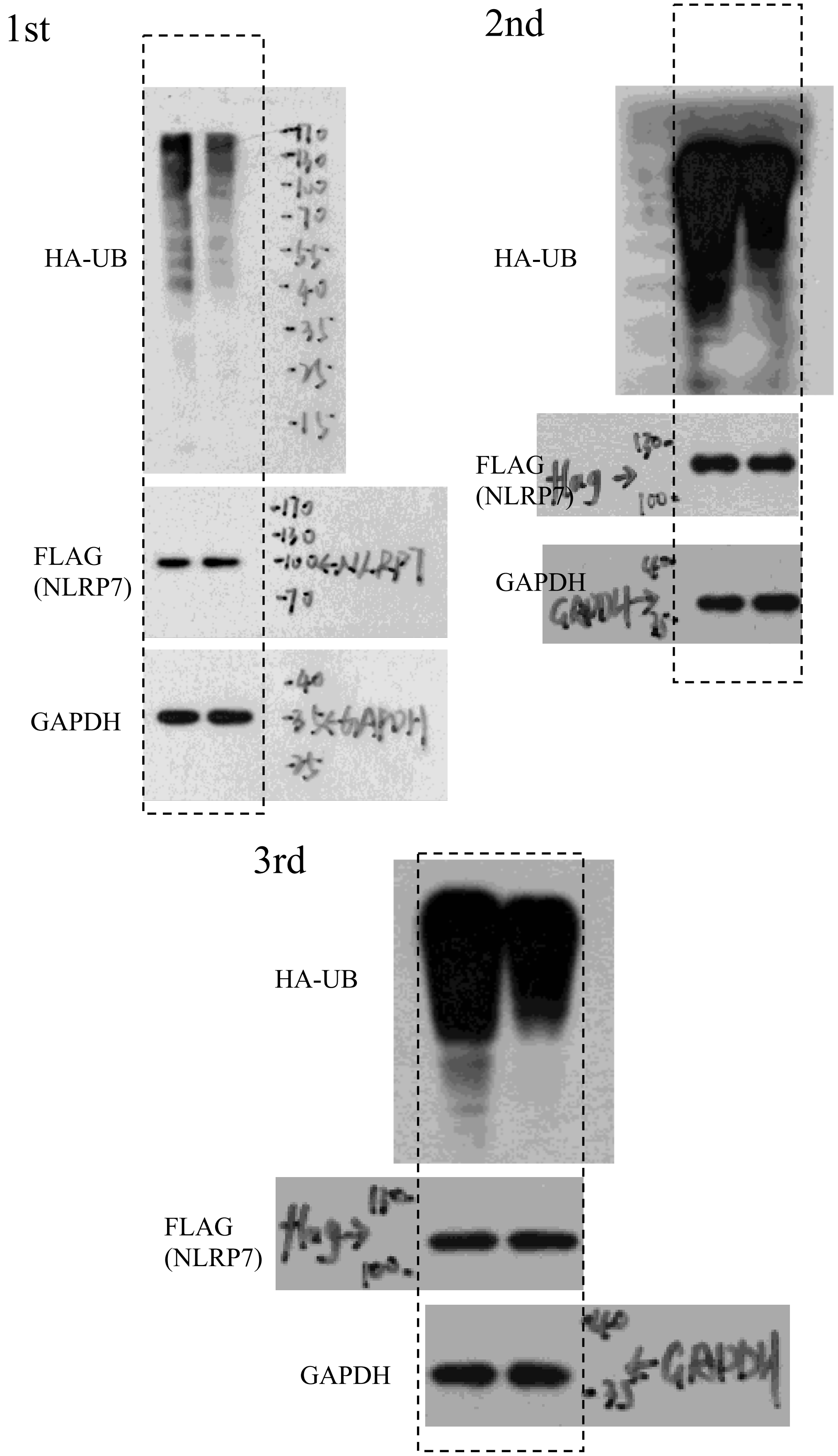
1st
HA-UB
FLAG
(NLRP7)
GAPDH
2nd
HA-UB
FLAG
(NLRP7)
GAPDH
3rd
HA-UB
FLAG
(NLRP7)
GAPDH

Figure 7A

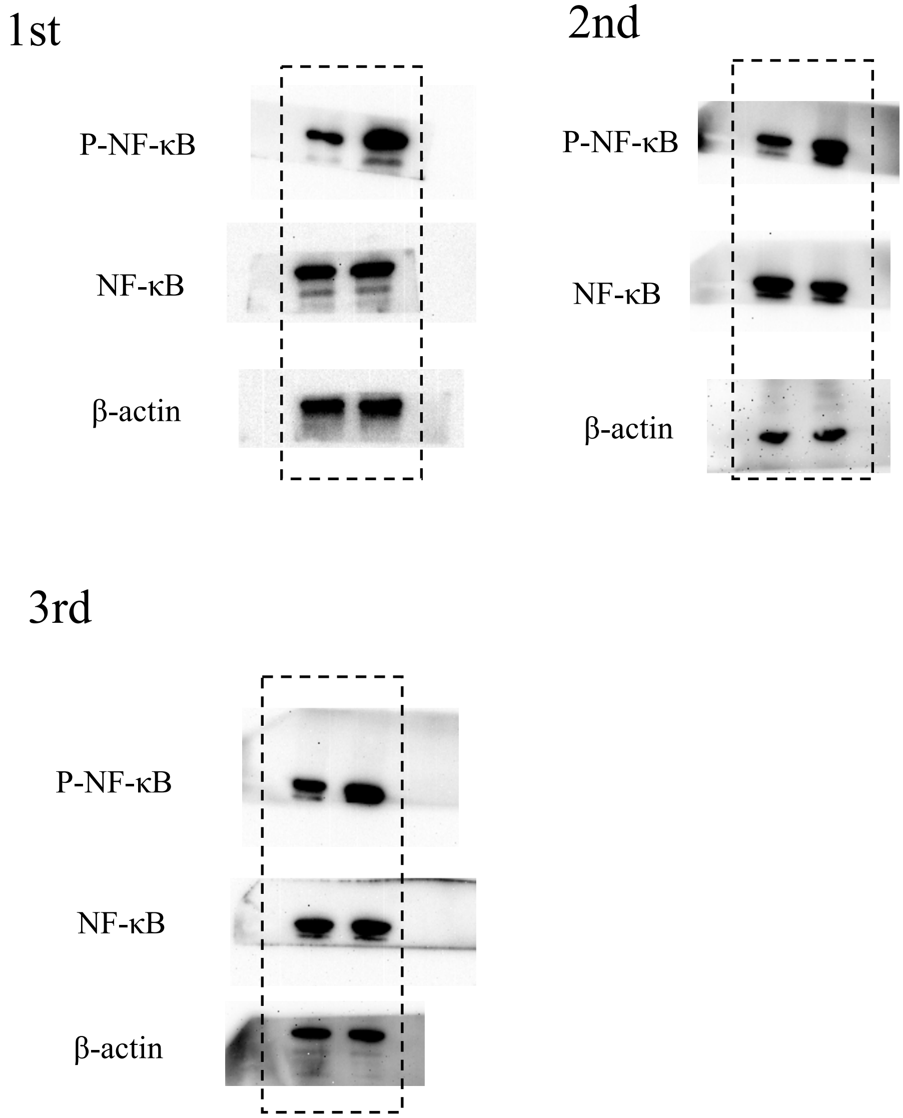
1st
P-NF-κB
NF-κB
β-actin
2nd
P-NF-κB
NF-κB
β-actin
3rd
P-NF-κB
NF-κB
β-actin
