# Supplementary figures and images for: NLRP7 deubiquitination by USP10 promotes tumor progression and tumor-associated macrophage polarization in colorectal cancer
Source: J Exp Clin Cancer Res. 2021 Apr 10;40:126. doi: 10.1186/s13046-021-01920-y (PMC8035766; doi:10.1186/s13046-021-01920-y)

Figure S1

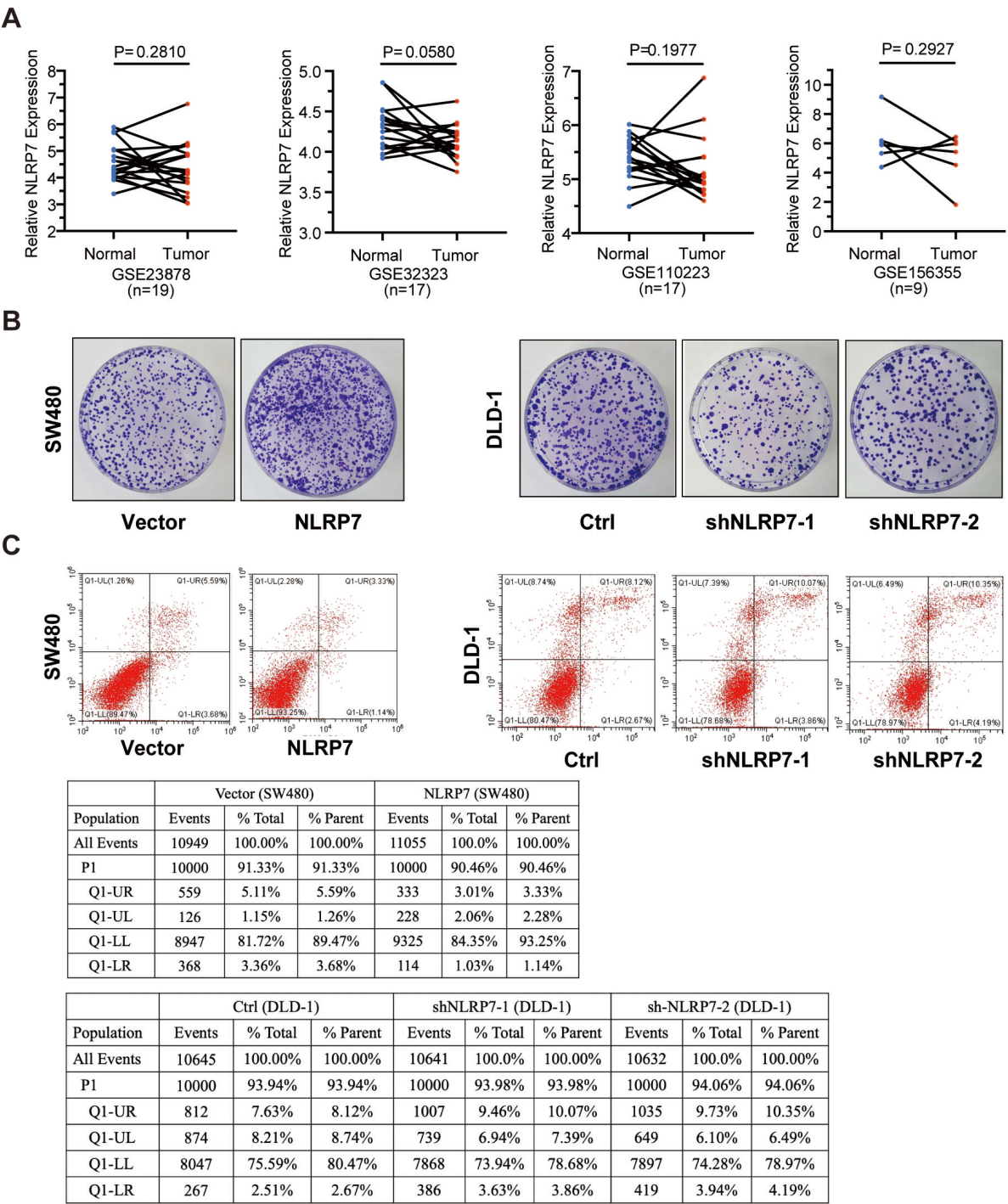

Figure S2

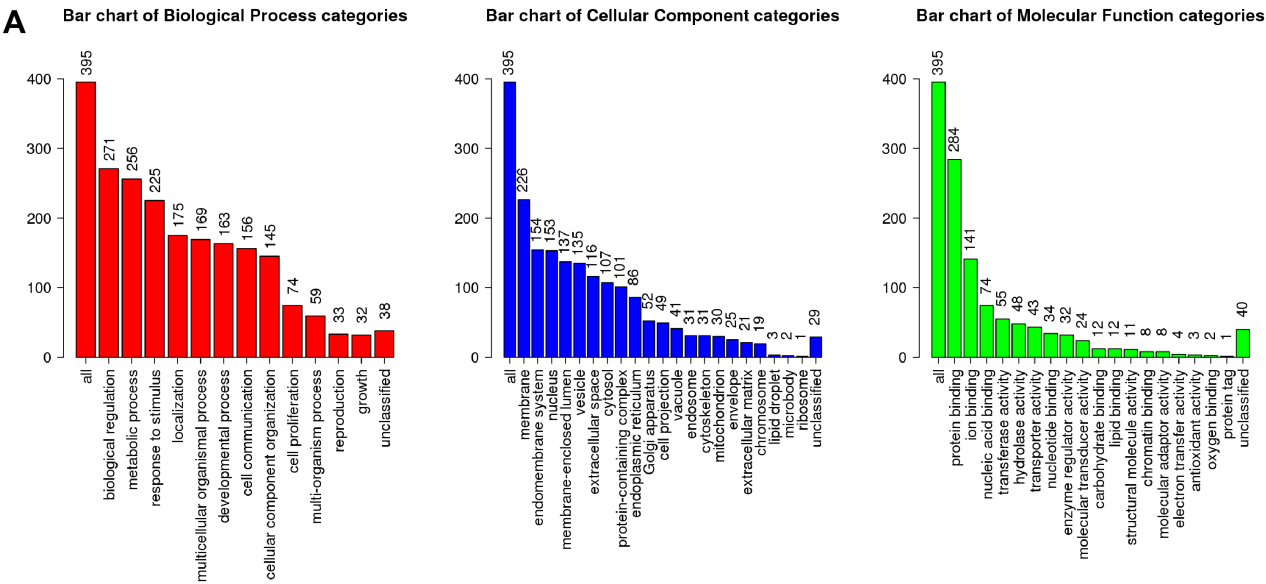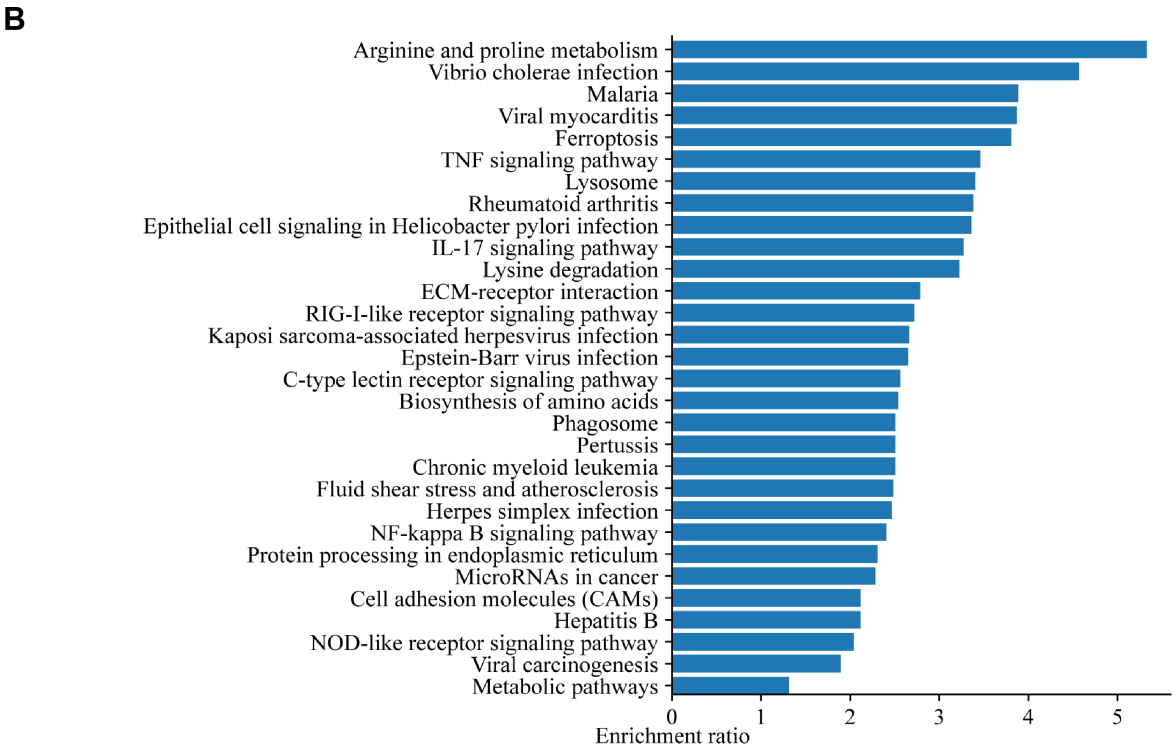

Figure S3

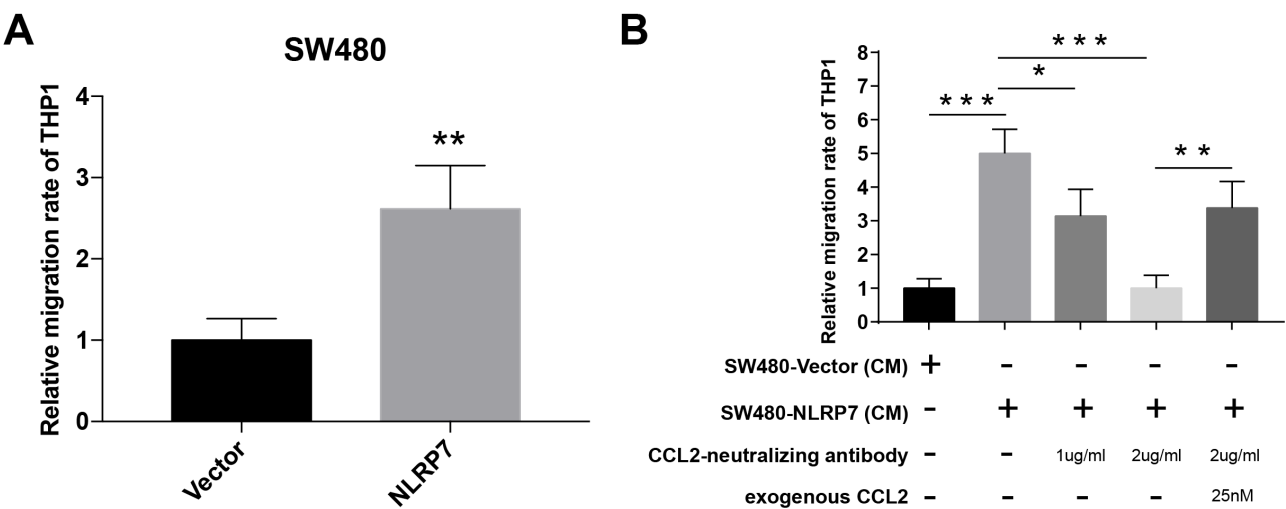

Supplement: Supplementary file 3 — Additional file 3: Figure S1. (A) Analysis of NLRP7 gene expression from the GEO dataset. (B) Representative images of colony formation assay. (C) Cell apoptosis in NLRP7 knockdown and overexpression CRC cells manifested by flow cytometry analysis. Figure S2. The GO (A) and KEGG (B) enrichment analysis results of 398 genes positively related with NLRP7 expression. Figure S3. (A) Chemotaxis of THP-1 cells treated with culture medium from cells overexpressing NLRP7. (B) CCL2-neutralizing antibody inhibited THP-1 cell chemotaxis, which was restored by exogenous CCL2. [file 13046_2021_1920_MOESM3_ESM.pdf]
